# Gre factors-mediated control of *hilD* transcription is essential for the invasion of epithelial cells by *Salmonella enterica* serovar Typhimurium

**DOI:** 10.1371/journal.ppat.1006312

**Published:** 2017-04-20

**Authors:** Tania Gaviria-Cantin, Youssef El Mouali, Soazig Le Guyon, Ute Römling, Carlos Balsalobre

**Affiliations:** 1 Departament de Genètica, Microbiologia i Estadística, Universitat de Barcelona, Barcelona, Spain; 2 Department of Microbiology, Tumor and Cell Biology, Karolinska Institutet, Stockholm, Sweden; Tufts University, UNITED STATES

## Abstract

The invasion of epithelial cells by *Salmonella enterica* serovar Typhimurium is a very tightly regulated process. Signaling cascades triggered by different environmental and physiological signals converge to control HilD, an AraC regulator that coordinates the expression of several virulence factors. The expression of *hilD* is modulated at several steps of the expression process. Here, we report that the invasion of epithelial cells by *S*. Typhimurium strains lacking the Gre factors, GreA and GreB, is impaired. By interacting with the RNA polymerase secondary channel, the Gre factors prevent backtracking of paused complexes to avoid arrest during transcriptional elongation. Our results indicate that the Gre factors are required for the expression of the bacterial factors needed for epithelial cell invasion by modulating expression of HilD. This regulation does not occur at transcription initiation and depends on the capacity of the Gre factors to prevent backtracking of the RNA polymerase. Remarkably, genetic analyses indicate that the 3’-untranslated region (UTR) of *hilD* is required for Gre-mediated regulation of *hilD* expression. Our data provide new insight into the complex regulation of *S*. Typhimurium virulence and highlight the role of the *hilD* 3’-UTR as a regulatory motif.

## Author summary

*Salmonella enterica* serovar Typhimurium is a foodborne pathogen that causes gastroenteritis in humans. To successfully trigger infection, *S*. Typhimurium invades epithelial cells, a process that requires the coordinated expression of a set of genes. HilD is a pivotal regulator of *S*. Typhimurium pathogenicity, as it activates the expression of the genes required for invasion of intestinal epithelium. Expression and activity of HilD are tightly regulated and respond to several environmental and physiological conditions. In this report, we introduce the transcription elongation as a novel level of regulation of *hilD*. We describe that the Gre factors, proteins that prevent backtracking of paused RNA polymerase complexes during transcription elongation, are required for the expression of HilD and the subsequent expression of genes involved in the invasion of epithelial cells.

## Introduction

*Salmonella enterica* serovar Typhimurium *(S*. Typhimurium*)*, an enteric bacterial pathogen that infects both humans and animals, is extensively used as a model organism in pathogenicity studies [1,2]. *S*. Typhimurium infection is asymptomatic in poultry, but causes gastroenteritis in humans [3]. Its infectious cycle is complex and requires the expression of a large number of virulence factors that are mostly encoded by chromosomal genes clustered in discrete regions known as *Salmonella* pathogenicity islands (SPIs). SPIs have been acquired through different evolutionary processes via horizontal gene transfer, with the successive acquisition of different genetic elements playing a determinative role in host adaptation [4]. Comparative genomic studies identify up to 21 SPIs in the *S*. Typhimurium genome, SPI-1 and SPI-2 being the best characterized [5]. SPI-1 contains genes required during the first steps of epithelial cell infection, while SPI-2 encodes genes needed for *S*. Typhimurium survival and replication inside host cells [6]. SPI-1 and SPI-2 genes encode their respective type three secretion systems (TTSS), TTSS-1 and TTSS-2, secreted effector proteins and regulators that coordinate the optimal expression of virulence genes [7–9]. In SPI-1, the HilA protein directly regulates the expression of secretion machinery components and various TTSS-1 effectors (S1 Fig) [10]. Moreover, HilA induces the expression of the regulator InvF, a transcriptional activator of *sic/sip* operons, encoding effector proteins [11,12]. HilA transcriptional expression is autoregulated and tightly modulated by the combined action of three AraC-like transcriptional activators: HilC, HilD and RtsA [13,14]. Each of these three regulators are positively autoregulated and can induce the expression of the other two, producing a positive feed-forward loop that controls SPI-1 gene expression [15]. HilD plays a major role in regulating *hilA* expression. Its expression and activity is targeted by many signaling pathways, with HilD acting as a hub that integrates diverse environmental and physiological cues to trigger *S*. Typhimurium invasion of epithelial cells [16]. Of note, HilD-mediated regulation is not restricted to the SPI-1 genes, as HilD also modulates the expression of genes located outside this genetic locus such as *sopE*, which encodes an effector protein secreted via TTSS-1, and *ssrAB*, which encodes the two-component system that acts as the central positive regulator of the SPI-2 genes [10,17,18]. Therefore, HilD plays a key role in *S*. Typhimurium pathogenicity.

Transcription, the first step in gene expression, is tightly regulated. Regulation of transcription initiation is crucial in determining the genomic response to physiological and environmental signals. Likewise, regulation during transcription elongation and termination has a pronounced effect on the steady state expression levels of particular genes and regulons, but is less well understood [19]. Brief transcriptional pauses in RNA polymerase (RNAP) activity that occur during elongation can quickly resolve spontaneously; however, sustained pauses may cause backtracking of the transcription elongation complex. In *E*. *coli*, the Gre factors, GreA and GreB, resolve backtracked complexes by interacting with the secondary channel of the RNAP and inducing endoribonuclease activity. These actions restore the proper positioning of the 3’-end of the nascent transcript within the RNAP active center [20,21]. Transcriptional pauses may act as regulatory events that affect the expression levels of specific genes [22]. In addition to their role in suppressing transcriptional pauses, Gre factors stimulate RNAP promoter escape and enhance transcriptional fidelity [23]. Gre factors occur widely in prokaryotes. The presence of two distinct clades among the Gre family members sharing high structural and functional homology, GreA and GreB, has been detected among proteobacteria. Members of the family outside proteobacteria resemble GreA more closely than GreB [24]. In *S*. Typhimurium, GreA and GreB share 34% identity and 57% similarity, numbers which are nearly identical to those observed in *E*. *coli* (35% and 56%, respectively).

In this report, we explored whether Gre factors are relevant in regulating pathogenicity in *S*. Typhimurium. We found that the Gre factors are required for the proper expression of the SPI-1 effector proteins and subsequent cell invasion as well as organ colonization in a mouse model of systemic infection. The dissection of the regulatory pathway let us conclude that Gre factors are essential for the expression of HilD, a major regulator of SPI-1 genes. Regulation of *hilD* does not occur at transcription initiation, but the 3’-untranslated region (UTR) is required for Gre-mediated regulation of *hilD* expression. This suggests that regulation depends on the ability of Gre factors to prevent backtracking of paused RNA polymerase complexes possibly coupled with downstream events. Our data provide new insights into the complex regulation of *S*. Typhimurium virulence and the role of the 3’-UTR of *hilD* as a regulatory motif.

## Results

### *S*. Typhimurium invasion of epithelial cells is impaired in bacterial cells lacking *greA* and *greB*

**Fig 1:**
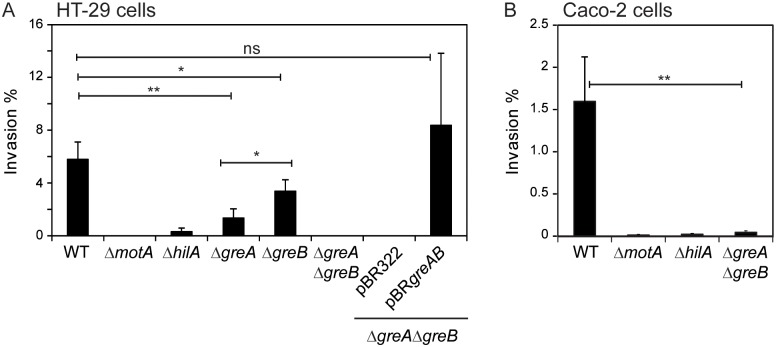
Invasion of epithelial cells by *S*. Typhimurium is impaired in strains deficient for the Gre factors. Cultures of the WT (SV5015) and the Δ*greA*, Δ*greB* and Δ*greA*Δ*greB* derivatives were assessed for invasion of HT-29 (A) and Caco-2 (B) cell lines. As a control, cultures of the invasion impaired mutants Δ*hilA* and Δ*motA* were used. A bar shows the arithmetic mean of experimental results and the error bar indicates the standard deviation. Significance was tested by an unpaired two–sided Student’s t-test. Statistical significance is indicated by *p<0.05, **p<0.01, ns: non-significant.

To elucidate whether Gre factors regulate *S*. Typhimurium pathogenicity, invasion assays were performed using strains deleted for *greA* and/or *greB*. The HT-29 epithelial cell line was infected with the wild-type (WT) SV5015 strain, the Δ*greA* strain lacking GreA, the Δ*greB* mutant deficient in GreB, and the double mutant Δ*greA*Δ*greB* strain lacking both Gre factors. The Δ*motA* and Δ*hilA* derivatives, deficient in invasion, were used as controls [25,26]. Determination of the percentage of intracellular bacterial cells after one hour of infection showed that, as expected, the WT strain did invade epithelial cells, whereas the Δ*motA* and Δ*hilA* derivatives showed impaired invasion (Fig 1A). Although the Δ*greA* and Δ*greB* mutants were invasive, they displayed only 23% and 58% of WT invasiveness, respectively. Remarkably, invasion was abolished in the strains lacking both GreA and GreB. To confirm that the inability to invade was caused by the absence of the Gre proteins, *trans*-complementation of the Δ*greA*Δ*greB* double mutant with a pBR322-based plasmid containing the *greA* and *greB* genes (pBR*greAB*) restored invasiveness. To validate the invasion defect mediated by Gre deficiency, a different strain and cell line background was used. Similar results were obtained when the effect of the Gre factors on invasion was also tested in HT-29 cells using virulent *S*. Typhimurium strain ATCC14028 and when performed with Caco-2 cells (S2 Fig and Fig 1B).

To corroborate the *in vitro* defect in epithelial cell invasion and to address the biological significance of the Gre factors in *Salmonella* pathogenesis, the potential of the Gre-deficient strain to cause systemic infection in *Salmonella* susceptible mice was investigated (Table 1). The bacterial load in livers and spleens of BALB/c mice (n = 5) inoculated orally with a 1:1 mixture of the WT and the Δ*greA*Δ*greB* strain was estimated four days after infection. While the WT strain was able to colonize the internal organs, the Δ*greA*Δ*greB* mutant was unable to do so, only being recovered from liver and spleen of one mouse (1000-fold lower than the wild type). Thus, our *in vivo* data highlight the biological significance of the Gre proteins in *Salmonella* pathogenesis.

**Table 1:** Competition assay of the Δ*greA*Δ*greB* mutant strain versus WT. Amount of bacteria in liver and spleen was determined at 4 days post infection. A total of ~2E+7 colony forming units (cfu) of WT and the Δ*greA*Δ*greB* strain at a 1:1 ratio was administered orally to 5 mice.

Mice	Liver (CFU/g)			Spleen (CFU/g)		
	WT	Δ*greA*Δ*greB*	CI	WT	Δ*greA*Δ*greB*	CI
#1	1.8E+5	4.7E+1	4.0E-4	2.4E+6	1.6E+3	1.0E-3
#2	3.0E+4	n.d.	< E-4	3.5E+5	n.d.	< E-3
#3	1.9E+4	n.d.	< E-4	2.2E+5	n.d.	< E-3
#4	1.8E+4	n.d.	< E-4	2.2E+5	n.d.	< E-3
#5	1.7E+2	n.d.	-	3.3E+2	n.d.	-

n.d.: non-detected

Invasion of epithelial cells requires a battery of effector proteins encoded mainly by genes in SPI-1 that are secreted through the TTSS encoded by the same genetic locus. Therefore, our results suggest that Gre factors are involved in the efficient expression of SPI-1 genes and that their absence elicits the avirulent phenotype of the Δ*greA*Δ*greB* strain.

### Gre factors are required for the expression of SPI-1 effector proteins

**Fig 2:**
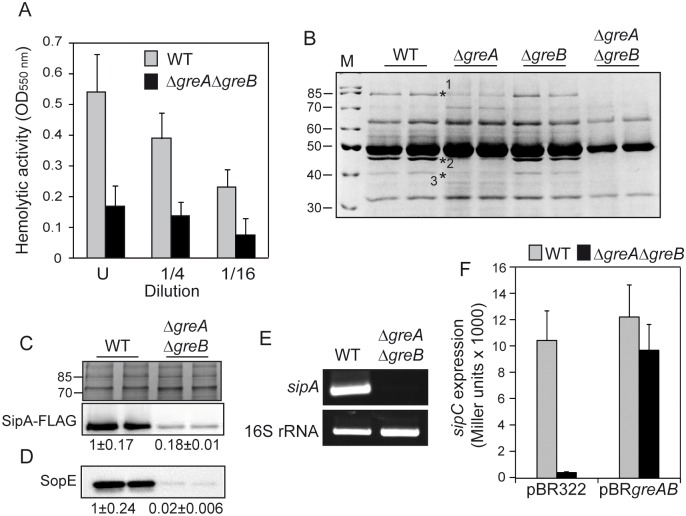
The Gre factors affect the expression of SPI-1 effector proteins. (A) Contact haemolysis assays with cell-free supernatants from cultures of the WT and Δ*greA*Δ*greB* derivative strains. The haemolytic activity of LB bacterial cultures of WT and Δ*greA*Δ*greB* strains was monitored as an increase in the OD_550nm_ of the supernatant of a blood suspension with serial dilutions of cell-free supernatants. U: undiluted supernatant. Three independent bacterial cultures were tested. (B) Cell-free supernatants of two independent LB cultures of WT (SV5015) and its Δ*greA*, Δ*greB* and Δ*greA*Δ*greB* derivatives. Extracts were analyzed by Coomassie blue stained 12.5% SDS-PAGE. Lane M: molecular mass markers (size in kDa indicated). The bands labelled were identified as SipA (1), FliD (2) and SipC (3) by LC-MS/MS. (C) Immunodetection (lower panel) of the SPI-1 encoded SipA-FLAG protein in whole culture extracts from two independent cultures of WT and Δ*greA*Δ*greB* derivative strains. The upper panel is a section of a Coomassie stained gel as a loading control. (D) Immunodetection of SopE protein was performed in extracts from WT and Δ*greA*Δ*greB* strains obtained from cell-free supernatants of two independent LB cultures. (E) Semiquantitative RT-PCR of *sipA* in total RNA samples from LB cultures of the WT (SV5015) and Δ*greA*Δ*greB* strains. 16S RNA was used as endogenous control to confirm that equivalent quantities of templates were used. (F) *sipC* transcriptional expression was tested in cultures of WT and Δ*greA*Δ*greB* derivative strains carrying a chromosomal *sipC*::*lacZ* fusion and either pBR322 or pBR*greAB*. A bar shows the arithmetic mean of experimental results and the error bar indicates the standard deviation from three biological replicates. All cultures were grown in LB at 37°C with vigorous shaking (200 rpm) up to an OD_600nm_ of 2.0.

Translocation of specific SPI-1-encoded effector proteins, such as the Sip proteins, has been associated with hemoglobin release during *S*. Typhimurium infection of erythrocytes *in vitro* [27]. Contact-dependent hemolysis was monitored with cultures of WT and Gre-deficient strains. The Δ*greA*Δ*greB* mutant could not lyse erythrocytes when compared to the parental strain (Fig 2A). Secreted proteins from WT, Δ*greA*, Δ*greB* and Δ*greA*Δ*greB* strains grown under conditions that induce invasiveness (cultivated in LB broth at 37°C with aeration to the early stationary phase (OD_600nm_ of 2.0), [18]) were analyzed by SDS-PAGE and Coomassie staining. Comparison of the secreted protein profiles (Fig 2B) indicated that: (i) extracts from the Δ*greA* mutant strain showed a significant decrease in the intensity of three protein bands (labeled with an asterisk in the Figure); (ii) extracts from the Δ*greB* mutant strain were apparently identical to those from the WT strain; and (iii) extracts from the Δ*greA*Δ*greB* mutant showed an overall decrease in protein secretion including the three above mentioned protein bands. To determine if these protein bands corresponded to SPI-1 effector proteins, extracts from WT and a Δ*hilA* strain were compared (S3 Fig). Since HilA is a transcriptional regulator that controls the expression of SPI-1 genes, the Δ*hilA* mutant is phenotypically equivalent to a deletion of the entire SPI-1 locus [15]. Interestingly, the three major protein bands that were missing in the extracts from the Δ*greA*Δ*greB* mutant were also absent in the Δ*hilA* extracts. LC-MS/MS (Liquid chromatography-mass spectrometry) revealed that the protein bands corresponded to the SPI-1 effector protein SipA, the flagellar cap protein FliD and the SPI-1 effector protein SipC.

These results are in agreement with the different levels of invasiveness presented by the mutant strains and indicate that Gre factors are required for either the expression or secretion of SPI-1 effector proteins. The amount of a SipA-FLAG tagged protein from whole culture extracts (cellular + extracellular proteins) was measured by immunodetection (Fig 2C). There was a significant drop (5.5-fold) in the total production of SipA in the Δ*greA*Δ*greB* strain compared to WT, demonstrating that Gre factors are required for the optimal expression of SPI-1 effector proteins. Expression of the SopE protein, encoded outside SPI-1 but secreted through TTSS-1, is co-regulated with the SPI-1-encoded effector proteins [10]. The amount of SopE secreted by the Δ*greA*Δ*greB* strain was diminished >25-fold when compared to WT (Fig 2D), demonstrating a crucial role of Gre factors in modulating SopE expression.

To determine the level at which Gre factors affect SPI-1 gene expression, transcriptional studies were performed. Semi-quantitative RT-PCR indicated a significant decrease in *sipA* mRNA steady state levels in the Δ*greA*Δ*greB* strain compared to WT (Fig 2E). This was corroborated using a chromosomal *sipC*::*lacZ* fusion, which demonstrated that there was a 10-fold reduction in *lacZ* expression in the Δ*greA*Δ*greB* strain compared to WT. This transcriptional phenotype can be complemented in the presence of pBR*greAB* (Fig 2F).

### Transcriptional expression of the major SPI-1 regulator HilA is impaired in strains lacking Gre factors

**Fig 3:**
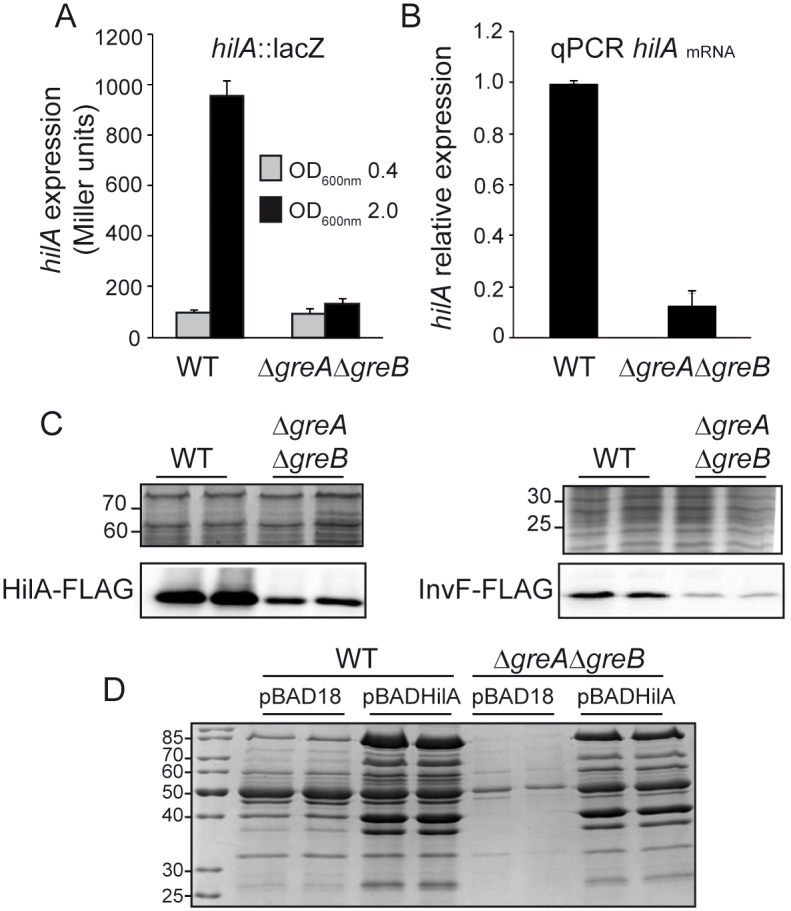
Gre factors are essential for HilA expression. (A) Transcriptional expression of *hilA* in WT and Δ*greA*Δ*greB* derivative strains. β-galactosidase activity from a *hilA*::*lacZ* fusion was assessed in LB cultures grown at 37°C up to logarithmic (OD_600nm_ 0.4) and stationary growth phase (OD_600nm_ 2.0). (B) Relative *hilA* mRNA quantification by qPCR in WT and Δ*greA*Δ*greB* derivative strains. Results are normalized with *gapA* (GAPDH) as an endogenous control. RNA samples were extracted from cultures of WT and Δ*greA*Δ*greB* derivative strains grown in LB at 37°C up to an OD_600nm_ 2.0. In A and B, a bar shows the arithmetic mean of experimental results and the error bar indicates the standard deviation from three biological replicates. (C) Immunodetection of HilA-FLAG (lower left panel) and InvF-FLAG (lower right panel) proteins in whole cell extracts from cultures of WT and Δ*greA*Δ*greB* derivative strains grown as in B. The upper panels are sections of Coomassie stained gels as loading controls. (D) Cell-free supernatants of LB cultures, grown at 37°C up to an OD_600nm_ of 2.0, of WT and Δ*greA*Δ*greB* derivative strains carrying either pBAD18 or pBADHilA. Arabinose (0.02%) was added in all cultures. Extracts were analyzed by Coomassie blue stained 12.5% SDS-PAGE.

As the expression of several SPI-1 effector proteins depends on the presence of Gre factors, we hypothesized that Gre factors may be acting upstream in the regulatory pathway controlling SPI-1 expression. The expression of most SPI-1 genes requires the transcriptional activator HilA, encoded in the SPI-1. HilA binds to target promoters to induce the expression of many genes involved in TTSS biogenesis, including *invF*, which promotes the subsequent expression of the *sic*/*sip* genes (reviewed by [26]). The effect of Gre factors on *hilA* transcriptional expression was studied using a chromosomal *hilA*::*lacZ* fusion (Fig 3A). Consistent with previous reports, *hilA* expression was induced in the WT strain in cells entering the stationary phase [18]. By contrast, *hilA* transcriptional expression was impaired in the Δ*greA*Δ*greB* mutant, suggesting that Gre factors are required for HilA transcriptional expression. This was further corroborated by qPCR analysis of *hilA* transcript levels, which showed a 10-fold drop in the Δ*greA*Δ*greB* strain compared to WT (Fig 3B). The effect of Gre factors on *hilA* transcriptional expression was also observed when looking at the levels of HilA-FLAG (Fig 3C). Accordingly, the expression of InvF, the AraC activator that is directly activated at the transcriptional level by HilA, was significantly reduced in the Δ*greA*Δ*greB* strain compared to WT (Fig 3C).

Our data show that Gre factors are involved in the transcriptional expression of both *hilA* and HilA-regulated genes. We aimed to determine whether Gre factors are required directly for the efficient transcription of genes encoding effector proteins or whether their effects are mediated only through regulating *hilA* expression. HilA expression was ectopically induced and its effect on effector protein production monitored in WT and the Δ*greA*Δ*greB* double mutant. As seen in Fig 3D, *hilA* ectopic expression elicited effector protein expression independently of Gre factors, suggesting that Gre factors are not essential for the transcription of the effector protein genes, but are required for regulating HilA expression or other regulator(s) that modulate *hilA* expression.

### Gre factors affect SPI-1 gene expression by modulating *hilD* expression

**Fig 4:**
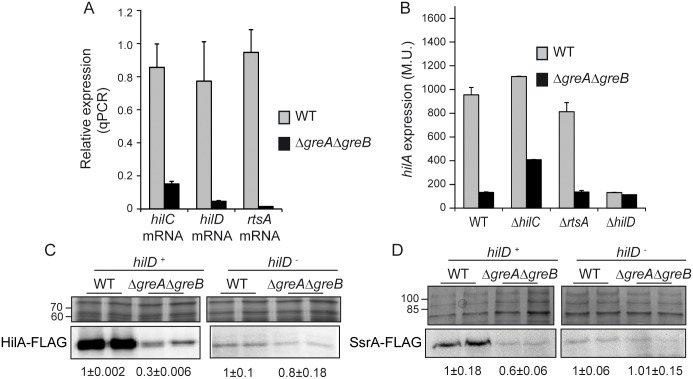
Gre factors-mediated regulation of *S*. Typhimurium virulence is focused in the regulation of the master regulator HilD. (A) Relative *hilC*, *hilD and rtsA* mRNA quantification by qPCR in WT and Δ*greA*Δ*greB* derivative strains. Results are normalized after detection of *gapA* (GAPDH) that was used as an endogenous control. Same RNA samples as in Fig 3B. (B) Transcriptional expression of *hilA* in WT, *hilC*, *rtsA* and *hilD* derivative strains either proficient (grey bars) or deficient (black bars) in the Gre factors was monitored by β-galactosidase activity determination from a *hilA*::*lacZ* fusion. In A and B, a bar shows the arithmetic mean of experimental results and the error bar indicates the standard deviation from three biological replicates. Immunodetection (lower panels) of HilA-FLAG protein (C) and the SPI-2 encoded SsrA-FLAG protein (D) in whole cell extracts from cultures of WT and Δ*greA*Δ*greB* derivative strains in a *hilD*^+^ and *hilD*^-^ genetic backgrounds. The upper panels are sections of Coomassie stained gels as loading controls. In all cases bacterial cultures were grown in LB at 37°C up to an OD_600nm_ of 2.0.

The transcriptional expression of *hilA* is tightly modulated by three AraC activators, HilD, HilC and RtsA, which form an auto-inducing regulatory loop where each protein can activate its own expression, as well as expression of the other two regulators [15]. Genetic analyses indicated that Gre factors are required for the transcriptional expression of these three activators (Fig 4A). Expression of *hilA* was determined in the WT and the Δ*greA*Δ*greB* mutant as well as in combination with deletion of each of the three different activators (Fig 4B). Under the experimental conditions used, HilC and RtsA were not needed to achieve high *hilA* expression; however, HilD was essential since *hilA* expression was abolished in Δ*hilD* mutants regardless of the presence or absence of Gre factors. *Trans*-complementation of the Δ*hilD* mutant with a pBR322-based plasmid containing the *hilD* gene restored *hilA* expression (S4 Fig), confirming the lack of *hilA* expression to be caused by the Δ*hilD* mutation. The requirement of HilD for *hilA* expression was further studied by monitoring HilA-FLAG expression in the presence or absence of HilD (Fig 4C). A drastic drop in intracellular HilA-FLAG levels in the Δ*greA*Δ*greB* mutant can only be detected in the HilD^+^ strains. Altogether, these results suggest that HilD plays a pivotal role in activating *hilA* expression under the experimental conditions used. A central role of HilD in regulating SPI-1 gene expression has been previously described since many signaling pathways that influence SPI-1 expression converge in controlling HilD [28]. Additionally, HilD not only regulates bacterial entry into host cells, but also coordinates the bacterial response to the intracellular milieu by modulating the expression of *ssrAB*, which encodes the central positive regulator of the SPI-2 genes [17]. SsrA expression was evaluated in WT and the Δ*greA*Δ*greB* mutant (Fig 4D), with the latter showing lower SsrA levels. Under the conditions used, *ssrAB* expression depended strongly on HilD, since there was no expression in a *hilD-*deficient strain regardless of the presence or absence of Gre factors. Our results suggest that Gre-mediated regulation of *hilD* is not restricted to SPI-1, but also affects other genetic loci, having a pronounced effect on the expression of virulence traits in *S*. Typhimurium.

**Fig 5:**
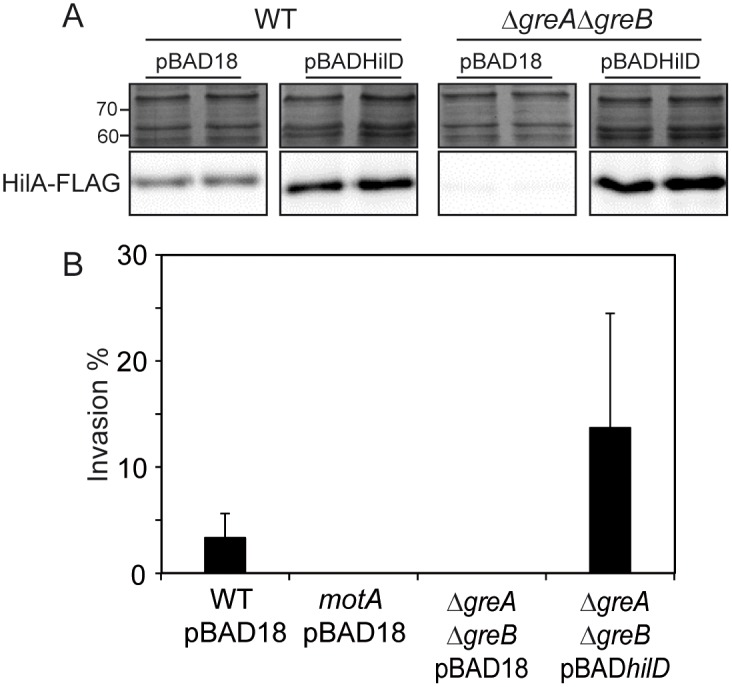
Overexpression of HilD restores HilA expression and epithelial cell invasiveness in Δ*greA*Δ*greB* strains. (A) In lower panels, immunodetection of the HilA-FLAG protein in whole cell extracts from cultures of WT and Δ*greA*Δ*greB* derivative strains carrying either pBAD18 or pBADHilD grown in LB at 37°C up to an OD_600nm_ of 2.0, arabinose (0.02%) was added in all cultures. The upper panels are sections of Coomassie stained gels as loading controls. (B) Invasion assays using WT, Δ*motA* and Δ*greA*Δ*greB* strains carrying the indicated plasmids. Bacterial cultures were grown as in Fig 1. A bar shows the arithmetic mean of experimental results and the error bar indicates the standard deviation.

We aimed to determine whether *hilA* downregulation and all the downstream effects are a consequence of Gre-mediated regulation of *hilD*. As shown in Fig 5A, ectopic induction of *hilD* expression elicited *hilA* expression even in the absence of Gre factors. Consistent with these results, the Δ*greA*Δ*greB* strain efficiently produced and secreted SPI-1 effector proteins and recovered the ability to invade epithelial cells when HilD expression was ectopically induced (S5 Fig and Fig 5B). Motility is required for effective invasion by *Salmonella* [25]. Motility, though, moderately 2-fold downregulated in the absence of Gre factors, was not restored upon overexpression of HilD (S6 Fig). Thus, Gre factors specifically regulate SPI-1 expression and epithelial invasiveness via *hilD* expression.

### The 3’-UTR of *hilD* is required for Gre-mediated regulation of *hilD* expression

**Fig 6:**
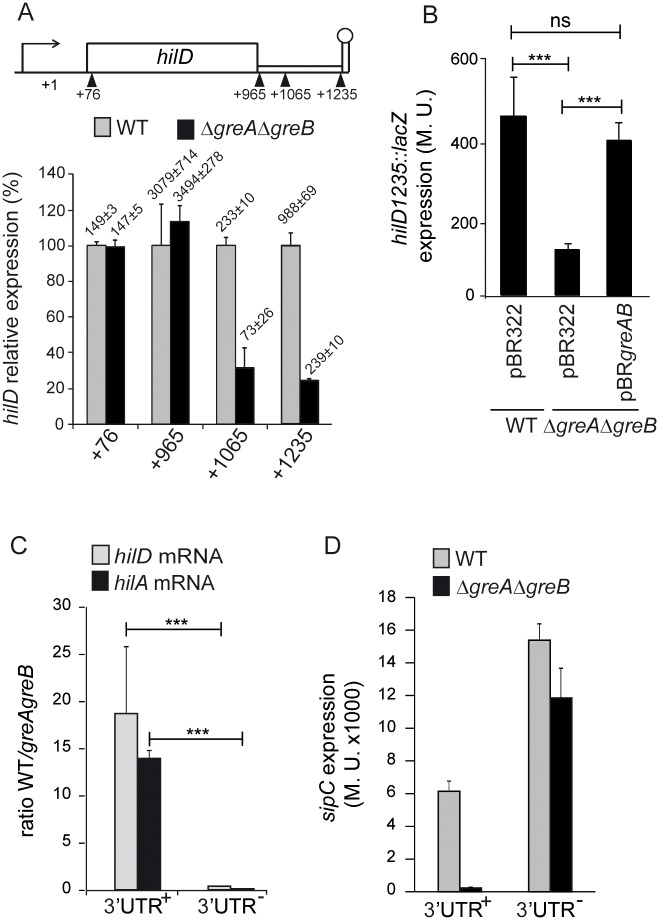
The 3’UTR of *hilD* is required for the Gre-mediated regulation. (A) Transcriptional expression from *hilD*::*lacZ* chromosomal fusions at positions +76, +965, +1065 and +1235 (relative location in the *hilD* gene is indicated in the upper panel). β-galactosidase activity was monitored in LB cultures grown at 37°C up to an OD_600nm_ of 2.0.of both WT and Δ*greA*Δ*greB* strains carrying the indicated fusions. (B) *hilD*_*1235*_::*lacZ* transcriptional expression was tested in cultures of WT and Δ*greA*Δ*greB* derivative strains carrying the indicated plasmids. β-galactosidase activity was determined as in A. (C) Ratio in the levels of *hilD* and *hilA* transcripts between WT and Δ*greA*Δ*greB* strains in *hilD* 3’UTR+ and *hilD* 3’UTR- genetic backgrounds. mRNA levels were monitored by qPCR using detection of *gapA* (GAPDH) as an endogenous control. Total RNA was isolated from cultures grown as in A. (D) Transcriptional expression from a *sipC*::*lacZ* chromosomal fusion. β-galactosidase activity was monitored as in A in cultures of both WT and Δ*greA*Δ*greB* strains in both *hilD*3’UTR^+^ and *hilD*3’UTR^-^ genetic backgrounds. In all panels, a bar shows the arithmetic mean of experimental results and the error bar indicates the standard deviation from three biological replicates. In B and C, to assess differences in the values, an unpaired, two-sided Student’s test was performed. Statistical significance is indicated by ***p<0.001, ns: non-significant.

Several regulatory mechanisms acting at the transcriptional, post-transcriptional, translational and post-translational levels of HilD expression have been described. It should be noted that in contrast to the qPCR data (Fig 4A), there was no differences in *hilD* transcriptional expression between WT and the Δ*greA*Δ*greB* mutant when a chromosomal transcriptional *hilD*::*lacZ* fusion at position +76 (41 bp within the *hilD* open reading frame (ORF)) was assessed (Fig 6A). This discrepancy indicates that Gre factors modulate *hilD* transcriptional expression after initiation of transcription. Other features that need to be taken in account when assessing *hilD* transcription are the presence of an intact *hilD* ORF, as the HilD protein positively regulates its own expression [15] and the presence of the newly described regulatory motif *hilD* 3’-UTR, that modulates *hilD* mRNA stability by promoting its rapid degradation [29]. Both regulatory units, the entire ORF and the 3’-UTR are absent in the *hilD*_76_::*lacZ* construct.

To assess the involvement of the *hilD* ORF and the 3’-UTR in Gre factor-mediated regulation of *hilD* two more constructs were investigated: i) the *hilD*_965_::*lacZ* fusion (at position +965, after the TAA codon of the *hilD* ORF, HilD^+^ 3’UTR^-^) and ii) the *hilD*_1235_::*lacZ* fusion (at position +1235, just upstream of the transcriptional terminator, HilD^+^ 3’UTR^+^) (Fig 6A). When comparing β-galactosidase activity levels among the different fusions in the WT strain, the expression of *hilD*_965_::*lacZ* was higher than that of *hilD*_76_::*lacZ*. The production of HilD from the *hilD*_965_::*lacZ* fusion and the subsequent positive *hilD* autoregulation may explain this result. Moreover, *hilD*_1235_::*lacZ* showed lower transcriptional activity than *hilD*_965_::*lacZ*. This result is consistent with the assigned role of the 3’-UTR in promoting mRNA degradation.

Next, the transcriptional expression of *hilD* in the WT and its Δ*greA*Δ*greB* double mutant was compared. As for the *hilD*_76_::*lacZ*, no difference was detected with the fusion *hilD*_965_::*lacZ*. Remarkably, a clear drop in *hilD* expression in the absence of the Gre factors can only be detected with the *hilD*_1235_::*lacZ* fusion (Fig 6A). Confirming that downregulation was due to the absence of Gre factors, *hilD*_1235_::*lacZ* expression recovered to WT levels in the presence of the pBR*greAB* plasmid (Fig 6B). Since the absence of Gre factors had no effect on *hilD* expression when the 3’-UTR was not present (*hilD*_76_::*lacZ* and *hilD*_965_::*lacZ*), the 3’-UTR of *hilD* is therefore required for Gre-factor modulation of *hilD* expression. To narrow down the region, the effect of the Gre factors was localized within the first 100 nucleotides of the 3’ UTR with fusion *hilD*_1065_::*lacZ* (Fig 6A). Consistently, the first 100 nucleotides of the 3’ UTR of *hilD* are sufficient to modulate *hilD* expression [29].

To further assess the requirement of the 3’-UTR for Gre-mediated regulation of *hilD*, the mRNA levels of both *hilD* and *hilA* were monitored by qPCR in a strain carrying a deletion of the *hilD* 3’-UTR, UTR^-^, and compared to the WT, UTR^+^ background. The results are given as a transcript ratio of WT versus Δ*greA*Δ*greB* (Fig 6C). Interestingly, only in the presence of the 3’-UTR a relative drop in *hilD* and *hilA* expression in the Δ*greA*Δ*greB* strain can be detected and consequently the specific transcripts were 18- and 14-fold more abundant in WT as compared to the Gre-deficient strains. These results are in agreement with the data derived from the *hilD*::*lacZ* fusions (Fig 6A).

Furthermore, the transcriptional expression of *sipC* was monitored (Fig 6D). Consistent with the assigned role of the *hilD* 3’-UTR [29], the results revealed that the lack of the *hilD* 3’-UTR upregulates *sipC* expression in the presence and absence of Gre factors, with higher expression than in the WT strain containing 3’UTR^+^. These results were generalized by the assessment of secreted SPI-1 effector proteins (S7 Fig).

### GreA-mediated rescue of backtracked paused complexes during transcription is crucial in promoting *hilD* expression

**Fig 7:**
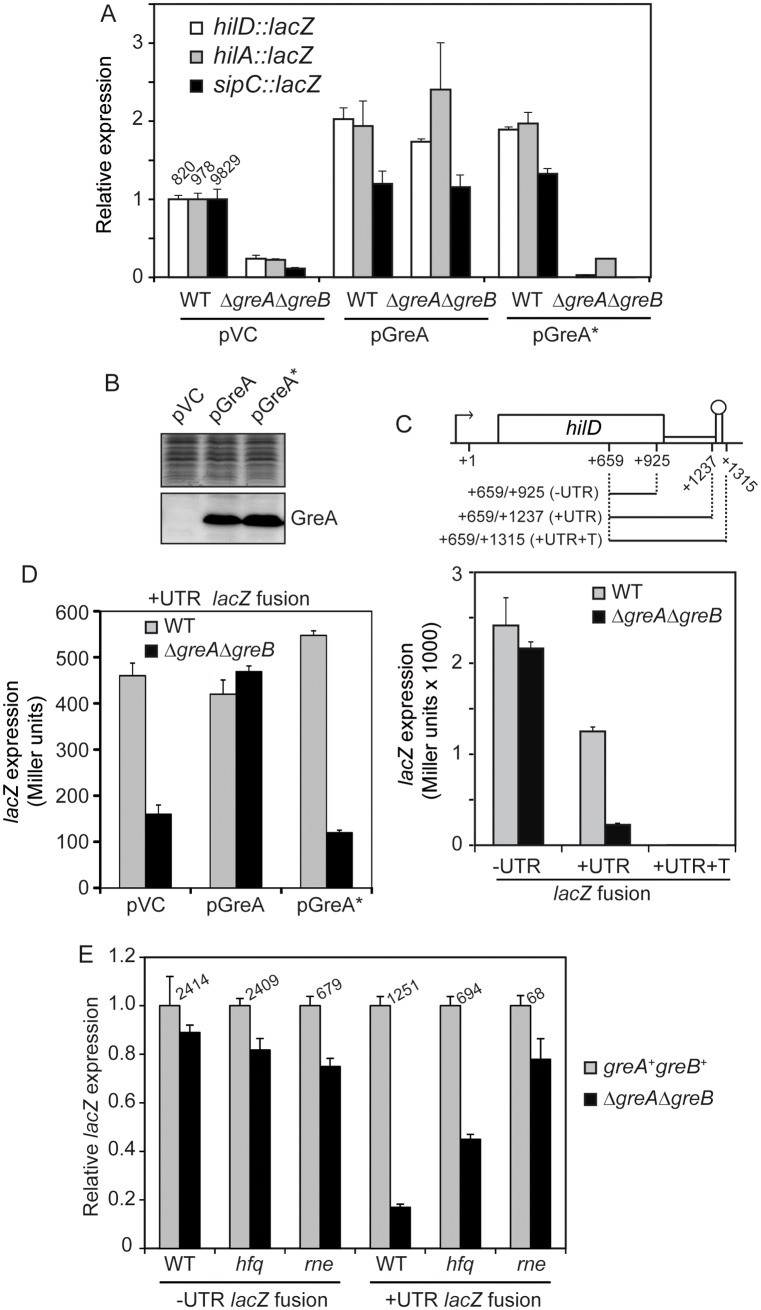
The anti-backtracking activity of the Gre factors is required for the expression of SPI-1 genes by alleviating a transcriptional pause located within the 3’-UTR of *hilD*. (A) Transcriptional expression from *hilD*:: *hilA*:: and *sipC*::*lacZ* chromosomal fusions. β-galactosidase activity was monitored in cultures of both WT and Δ*greA*Δ*greB* strains carrying the following plasmids pHM1883 (pVC), pHM1873 (pGreA) and pHM1854 (pGreA*). (B) Immunodetection of GreA protein was performed in extracts from the Δ*greA*Δ*greB* strain with plasmids as in A. (C) *lacZ* expression from plasmids constructs carrying the indicated *hilD* fragments (-UTR, +UTR and +UTR+T) cloned in pTT68 vector downstream of a P_BAD_ promoter and upstream of a promoter less *lacZ* gene in both WT and Δ*greA*Δ*greB* strains. (D) Expression from the +UTR *lacZ* fusion, as denoted in panel C, in WT and Δ*greA*Δ*greB* strains carrying the plasmids pHM1883, pHM1873 and pHM1854. (E) Effect of the Gre factors on expression of the -UTR and +UTR *lacZ* fusions, as denoted in panel C, in WT and its *hfq* and *rne*537 mutants. In all cases, cultures were grown in LB at 37°C up to an OD_600nm_ of 2.0. In B, D and E, 0.02% arabinose was added to the LB medium. In A and C-E panels, a bar shows the arithmetic mean of experimental results and the error bar indicates the standard deviation from three biological replicates.

In *E*. *coli*, the acidic residues D41 and E44 of the GreA protein are required to prevent backtracking of paused complexes thereby suppressing transcriptional pauses. A greA* (D41A, E44Y) mutant, devoid of the rescue function for transcriptional arrest, has been described previously [30]. The GreA protein of *E*. *coli* and *S*. Typhimurium are highly conserved, sharing 96.83% identity including the above mentioned acidic residues. We used the *greA* (WT) and greA* (D41A, E44Y) from *E*. *coli* to determine if the anti-backtracking activity of GreA is associated with the regulation of SPI-1 genes. As shown in Fig 7A, transcriptional expression of *hilD*, *hilA* and *sipC* was fully restored when the *greA* gene of *E*. *coli* was introduced into the *S*. Typhimurium Δ*greA*Δ*greB* double mutant. However, this was not observed with the greA* variant, indicating that the anti-backtracking activity of GreA is needed for the transcriptional expression of *hilD* and, consequently, SPI-1 genes. Immunodetection using a monoclonal anti-GreA antibody confirmed equal expression of *greA* and greA* in *S*. *typhimurium* (Fig 7B).

Our results suggest that Gre factors affect SPI-1 expression by a mechanism that includes prevention of a transcriptional arrest located in the *hilD* 3’-UTR. To explore the possible presence of a transcriptional arrest site within the 3’-UTR, we used an *in vivo* approach involving a pQF-50 based vector carrying a P_BAD_ promoter upstream of the promoter-less *lacZ* gene. Three different fragments of *hilD* were cloned between the P_BAD_ promoter and the *lacZ* gene (Fig 7C, upper panel): a 267-bp fragment from *hilD* encoding the sequence upstream of the 3’-UTR (+659/+925); a 579-bp fragment (+659/+1237) carrying the entire *hilD* 3’-UTR; and a 657-bp fragment (+659/+1315) carrying both the 3’-UTR and the native *hilD* transcriptional terminator downstream of the 3’-UTR. These constructs were introduced into *araBAD araC* SV5015 derivatives to increase sensitivity to arabinose induction and *lacZ* expression was monitored under permissive conditions (0.02% arabinose) in both WT and the Δ*greA*Δ*greB* mutant (Fig 7C). With the construct carrying the 267-bp fragment lacking the 3’-UTR (-UTR), the expression level was not affected by the presence or absence of Gre factors. For the construct carrying the 3’-UTR and the native terminator (+UTR+T), there was no β-galactosidase activity, demonstrating the effectiveness of the *hilD* Rho-independent transcriptional terminator. Remarkably, the absence of Gre factors had a clear effect when using the construct carrying the 3’-UTR, but lacking the terminator (+UTR). Expression decreased more than 5-fold in the absence of Gre factors, suggesting that during transcription of the 3’-UTR sequence an arrested transcription complex is formed which is released by the Gre factors. Consistently, expression of *lacZ* preceded by the +UTR raises to WT levels upon *greA* overexpression, whereas no recovery was detected with the greA* (D41A, E44Y) variant (Fig 7D).

The 3’-UTR of *hilD* plays a role in *hilD* mRNA turnover and in Hfq-mediated modulation of *hilD* expression [29]. To explore whether mRNA turnover and Hfq are involved in the Gre-mediated control of *hilD* expression, Δ*hfq* and *rne*537 mutations were generated in *greA*^+^*greB*^+^ WT and Δ*greA*Δ*greB* double mutant backgrounds. Expression of *lacZ* preceded by -UTR fragment was only moderately altered in the absence of Gre factors in WT, *hfq* and *rne*537 (Fig 7E), consistent with the fusion *hilD*_965_::*lacZ*, lacking the 3’-UTR, to be Gre factor independent (Fig 6A). In contrast, while expression of *lacZ* preceded by +UTR is 5.5-fold downregulated in the absence of the Gre factors, absence of an intact RNAseE reduced this difference to 1.3-fold suggesting that mRNA turnover plays a relevant role in Gre-mediated control of transcriptional regulation involving the +UTR. In the *hfq* genetic background a 2.2-fold drop was observed indicating a partial role for Hfq. This is consistent with mRNA turnover and Hfq-mediated regulation playing a relevant role in the control of genetic constructs containing the *hilD* 3’-UTR [29]. The Gre factors regulate expression when the 3’ UTR of *hilD* is present, presumably through its anti-backtracking activity. The molecular mechanism by which *hilD* expression is effectively downregulated in the absence of the Gre factors remains elusive, but RNA processing seems to play a crucial role.

## Discussion

Gre factors were initially described to prevent backtracking of paused complexes during transcription elongation thereby preventing transcriptional arrest, with further studies assigning additional roles in transcription initiation and fidelity [31]. Genes encoding Gre proteins are found in most bacteria. It has been reported that mutations in genes encoding Gre factors elicit hypersensitivity to ionic detergents, high temperatures and osmotic shock, suggesting that Gre factors are involved in adapting to harsh environments [32–34]. Interestingly, *greA* is a member of the sigma E regulon in both *E*. *coli* and *S*. Typhimurium, further indicating a potential role of GreA in cell stress response [35–37]. Moreover, *greA* expression is upregulated during the stress response to hypoxia and acid in *Mycobacterium avium* and *Streptococcus mutans*, respectively [38,39]. However, a direct relationship between the molecular mode of action of Gre factors during transcription and their physiological role in the cell is not clear. Few studies on the *in vivo* function of the Gre factors exist. In *E*. *coli* K12 strains only a discrete effect on the global gene expression pattern was detected when the level of GreA production was altered [23]. In this report, we demonstrate that Gre factors are required for the optimal expression of virulence factors in *S*. Typhimurium, since the ability to invade epithelial cells and produce SPI-1-encoded effector proteins was fully impaired in strains lacking both GreA and GreB. The absence of either GreA or GreB had partial effects compared to those elicited by the absence of both Gre factors. Moreover, the absence of only GreA had a greater impact on *S*. Typhimurium virulence than the absence of only GreB. This suggests that Gre factors might be functionally exchangeable up to a certain point, whereby GreA seems to play a more relevant role in *S*. Typhimurium pathogenesis than GreB. Similar observations on the functional redundancy of the Gre factors have also been described in *E*. *coli* [23,30].

**Fig 8:**
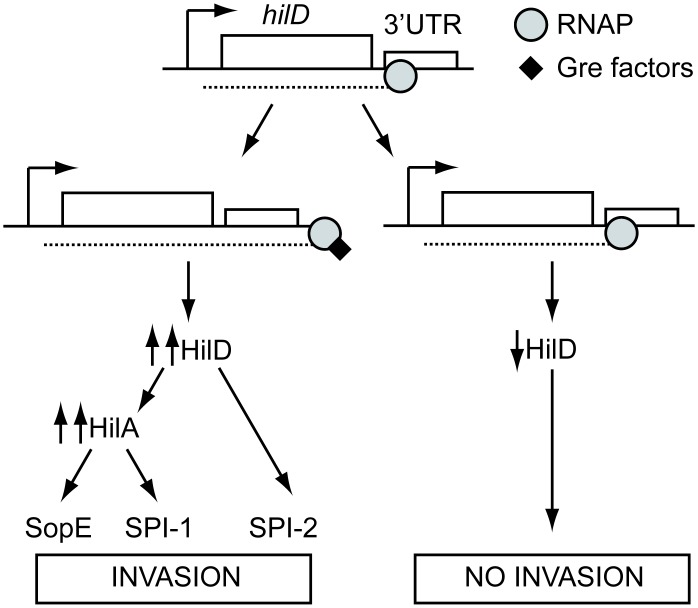
Summary of the effects of Gre factor deficiency in the expression of virulence in *S*. Typhimurium.

We observed that the major regulator of SPI-1, HilD, was indeed the target of Gre-mediated regulation. As summarized in Fig 8, Gre factors affect *hilD* expression during transcription elongation, targeting the recently described 3’-UTR regulatory motif of the *hilD* gene. Although there is currently no physical evidence for an arrested or backtracked complex during *hilD* transcription on which the Gre factors act, the requirement of the anti-backtracking activity of Gre factors suggest a model where Gre factors contribute to the regulation of *hilD* expression by rescuing an arrested or backtracked complex that occurs in the *hilD* 3’-UTR during transcription elongation (Fig 8). Further studies will be needed to address how backtracked RNAP complex potentially in combination with other mechanisms affects *hilD* expression.

HilD expression and activity are very tightly regulated, highlighting its pivotal role in the biology of *S*. Typhimurium. HilD acts as a regulatory hub for virulence coordinating the expression of several virulence factors encoded inside and outside SPI-1 [18,28,40–42]. Indeed, in this report we provide further corroboration of the role of HilD in regulating SPI-2 gene expression. Given the significant impact of HilD on gene expression in *S*. Typhimurium, HilD-mediated activation of gene expression only occurs when several environmental and physiological cues provide permissive conditions. Multiple signaling pathways control *hilD* transcription initiation [28]. CsrA post-transcriptionally regulates *hilD* [41]. At the post-translational level, factors such as HilE and the Lon protease affect HilD activity and stability, respectively, while different metabolites, including L-arabinose and fatty acids, have also been shown to modulate HilD activity [43–46]. In this report, we provide evidence that HilD regulation also occurs during transcription elongation in the 3’-UTR.

Pauses during transcription elongation can be targeted in gene expression regulation. In eukaryotes, paused complexes with RNAPII are affected by environmental cues. In bacteria, transcriptional pauses are involved in transcription attenuation, transcription termination and coupling transcription and translation [22]. Studies in *Streptococcus pneumoniae* suggest that transcription elongation is a highly regulated step of gene expression, whereby GreA plays a relevant role by preventing long-living pauses during transcription [47]. Regulatory pausing events are mainly localized in proximal promoter sequences, often in the 5’-UTR, but in *hilD*, it is the 3’-UTR that putatively contains a transcriptional pause. In bacteria, investigation of the features of 3’-UTRs of genes is a growing field of study, with the 3’-UTR possibly acting as a reservoir for sRNAs [48]. 3’-UTRs are rather abundant as in the *Staphylococcus aureus* genome, where up to a third of the genes carry long 3’-UTRs [49].

The *hilD* 3’-UTR is a regulatory sequence that overall affects transcript stability, since its absence increases *hilD* transcript levels. Furthermore, positive regulation of *hilD* expression by Hfq has been reported to require the 3’-UTR sequence [29]. As Hfq is a major sRNA chaperone, it probably regulates *hilD* expression through its 3’-UTR by a mechanism involving sRNA-based post-transcriptional regulation. In the present study, we propose that *hilD* expression is also regulated during transcription elongation of the 3’-UTR. Further studies are needed to elucidate whether changes in the kinetics of transcription elongation in the 3’-UTR affect *hilD* expression. Taking into consideration the described role of the *hilD* 3’-UTR, our results suggest that the anti-backtracking activity of Gre factors beyond *hilD* ORF transcription elongation may decrease susceptibility to mRNA degradation, possibly by stimulating the generation of certain RNA structures or promoting optimal interactions with sRNAs to protect the *hilD* transcripts from degradation. Thus, our report provides evidence for an additional mechanism of regulation of *hilD* expression at the post-transcriptional level.

## Materials and methods

### Bacterial strains, plasmids and growth conditions

Bacteria strains and plasmids used in this study are listed in S1 Table. Bacteria were grown in LB (5 g/L NaCl, 10 g/L tryptone, 5 g/L yeast extract), and LB agar plates with 15 g/L agar. Lac phenotype from strains with *lacZ* fusions was monitored on LB agar plates supplemented with X-gal (40 μg/mL). When needed, antibiotics were added at the following concentrations: chloramphenicol (Cm) 15 μg/mL, kanamycin (Km) 50 μg/mL, ampicillin (Amp) 50 μg/mL, streptomycin (Sm) 20 μg/mL and spectinomycin (Sp) 25 or 50 μg/mL. Arabinose 0.02% (w/v) was added to the cultures for induction of genetic constructs with the P_BAD_ promoter.

### Construction of mutants

Chromosomal mutants were generated by one step gene replacement by homologous recombination [50]. In general, entire open reading frames except 40 nucleotides at the beginning and at the end of the gene were replaced by either a kanamycin (Km) or chloramphenicol (Cm) resistance marker. The Km or Cm resistance gene along with target gene homologous overhangs was PCR-amplified from pKD4 or pKD3, respectively, and electroporated into *S*. Typhimurium carrying pKD46. Primers used in this work are listed in S2 Table. Recovered colonies were purified on LB medium containing the corresponding antibiotics. To obtain tagged proteins for immunodetection purposes, a 3×FLAG tag linked to a kanamycin cassette was amplified from pSUB11 plasmid [51] and inserted in frame upstream of the stop codon of the protein of interest. For transcriptional studies, *lacZ* fusions were constructed as described by Ellemeier et al [52]. Briefly, first the resistance cassette was removed expressing the FLP protein encoded in plasmid pCP20 that promotes site-specific recombination between the FRT sites. Next, either plasmid pKG136 or plasmid pKG137 were transformed into the resulting strains. Again, FLP-mediated recombination achieved from plasmid pCP20 results in an integrated *lac* fusion into the gene of interest.

Phage transduction of mutant alleles into a novel strain background was carried out with phage P22 HT/int4. Transductants were colony purified twice on EBU LB agar plates containing 0.25% (p/v) glucose, 0.25% (p/v) K_2_HPO_4_, 0.0125 g/L evans blue and 0.0250 g/L fluorescein [53]; and appropriate antibiotics. All constructed mutants were verified by PCR with control primers located in the genes flanking the deleted open reading frame.

### Plasmid construction

The *greA*, *greB* and *hilD* genes were cloned in pBR322 vector in order to perform complementation experiments. Primers greASalmUP/greASalmDOWN, greBSalmUP/greBSalmDOWN and hilDFw322/hilDRv322A were used to PCR amplify the different genes. After cloning the fragments in pGEM-T, either the *Eco*RI-*Bam*HI fragments (*greA* and *greB)* or the *Bam*HI-*Sal*I fragment (*hilD*) were cloned subsequently in pBR322, resulting plasmids pBR*greA*, pBR*greB* and pBR*hilD*. For genotyping purposes, the primers pBR-FW/pBR-RV were used. To generate the pBR*greAB* plasmid, primers greASalIUP and greABamHIDOWN, were used to amplify the *greA* gene. The PCR fragment was cloned into *Sal*I-*Bam*HI pBR*greB*. For genotyping the primers pBRFw/greASalIUP or greBSalmUP/greASalIUP were used.

The ORF of *hilD* was PCR amplified using the primers hilDBADFw and hilDBADRev and cloned in the *Eco*RI and *Xba*I sites of pBAD18. Different fragments of the *hilD* gene were cloned in the pTT68 vector, downstream of the arabinose inducible promoter P_BAD_ and upstream of a promoter less *lacZ* gene. The *hilD* fragments were PCR amplified with the primer pairs hilDNcoI1/UTRSalI3, hilDNcoI1/UTRSalI8 and hilDNcoI1/UTRSalI6. The *Nco*I-*Sal*I PCR fragments were cloned in the same restriction sites of pTT68. The generated plasmids were named pTTORF, pTT3’UTR and pTT3’UTR+T, respectively.

### Invasion assay

Invasion assays were performed as previously described [25]. The human colon adenocarcinoma cell lines HT-29 (ATCC HTB 38) and Caco-2 (ATCC HTB 37) were grown to confluence in 24-well plates in RPMI-1640 medium (Life Technologies) supplemented with 25 mM HEPES, 2 mM L-glutamine and 10% fetal calf serum (Hyclone) at 37°C in 5% CO_2_. *S*. Typhimurium was grown in LB containing 0.3 M NaCl in standing culture for 16h, diluted 1:100 in fresh medium and grown until OD_600nm_ of 0.6 at 37°C statically. Bacteria were washed with cold PBS and resuspended in RPMI-1640 medium. A 100 μL aliquot of the bacterial suspension were added to each well of confluent epithelial cells (MOI of 1.7, approximately). One hour post infection, supernatant was removed and RPMI-1640 medium containing gentamicin at a final concentration of 100 μg/mL was added to the cells for 1 h to kill remaining extracellular bacteria. Cells were gently washed twice with PBS and disrupted with 1% Triton X-100 (Sigma). The number of intracellular bacteria was determined by counting colony-forming units (CFU). The Δ*motA* and Δ*hilA* mutants were used as a negative control in all assays. The invasion percentage was calculated as CFU recovered inside cells after 1 h incubation with reference to the inoculum CFU at time of inoculation as 100%. Results are based on at least two biological replicates consisting of two technical replicates each.

### Animal studies in BALB/c mice

WT SV5015 and its Km-resistant Δ*greA*Δ*greB* derivative (TGC65) were grown in LB medium at 37°C up to an OD_600nm_ of 2.0. The bacteria were pelleted and diluted 1/10 in sterile PBS. The input ratio of a 1:1 mixture of WT and Δ*greA*Δ*greB* suspensions was determined by plating serial dilutions onto LB-Sm for total CFU and on LB-SmKm plates for estimation of CFU for the Δ*greA*Δ*greB strain*. A group of 5 8-week-old female BALB/c mice was infected orally with ~ 2E+7 CFU in 200 μL. Mice were sacrificed by CO_2_ asphyxiation four days post infection and liver and spleen were removed and homogenized. The homogenates were treated with sodium deoxycholate (0.01%), serially diluted and plated onto LB-Sm and LB-SmKm in triplicates. CFU/g were determined and CI values were calculated as the mean ratio of mutant versus WT CFU, normalized to the input ratio [54]. All animal care and handling were performed according to Federation of European Laboratory Animal Science Associations (FELASA) guidelines and were under the approval of the University of Barcelona (UB) Ethical committee.

### Secreted protein extracts

Protein extracts of the cell free supernatants, corresponding to the secreted protein fraction, was analyzed. Bacterial strains were cultured in LB medium at 37°C up to an OD_600nm_ of 2.0 (early-stationary phase). A 5 mL culture aliquot was centrifuged for 10 min at 4000 g at room temperature. The supernatant was filtered through a 0.22 μm filter and the proteins precipitated using trichloroacetic acid (TCA) [55]. When whole culture extracts were obtained, bacterial cultures (cells and medium) were TCA precipitated.

### SDS-PAGE and western blot analysis

Secreted protein extracts and whole cell extracts were separated on a 12.5% SDS-PAGE gel. To detect total protein, Coomassie staining was performed. When specific proteins were detected, proteins were electrotransferred onto a PVDF membrane (Bio Rad). For immunodetection the following antisera were used; monoclonal anti-FLAG (Sigma) polyclonal anti-SopE [56] and monoclonal anti-GreA (Neoclone). Detection was with a HRP-conjugated secondary antibodies and ECL Prime Western Blotting detection reagent (GE Healthcare). Visualization of the detected bands was performed using Molecular Imager ChemiDoc XRS System and Quantity One software (Bio Rad). Prior to western blot analysis from whole cell extracts, the protein content of samples normalized by OD_600nm_ (biomass of original cultures) was corroborated by Coomassie staining. Equally, loading of secreted protein extract was normalized to the culture biomass (OD_600nm_). Routinely, normalization by biomass of cultures grown to obtain secreted protein extracts was corroborated by SDS-PAGE and Coomassie staining of the cell extract from the cultures. Moreover, immunodetection of the cytoplasmic cyclic AMP receptor protein CRP was performed in secreted protein extracts to exclude contamination with cellular proteins. A representative control experiment performed on a secreted protein extract is shown in S8 Fig.

### Haemolytic activity

The haemolytic activity was determined as described [57]. Briefly, the strains were grown in LB medium at 37°C up to an OD_600nm_ of 2.0. An aliquot of 5 mL was centrifuged 15 min at 4000 g at room temperature, and the supernatant was filtered by 0.22 μm filter and kept on ice. The defribrinated sheep blood was centrifuged at 1500 g during 5 min at 4°C and the blood cells resuspended with cold PBS in order to eliminate debris from broken cells. This process was repeated as many times as required, until supernatant was transparent. In a 96-well plate, aliquots (50 μL) of different serial dilutions of the cell-free supernatant were mixed with 50 μL of defribrinated sheep blood stock solution. Next, mixtures were incubated statically at 37°C during 2.5 hours. After incubation, 150 μL of PBS were added to each well and the plates were centrifuged (10 min, 400 g, 4°C). A 100 μL aliquot of the supernatant was removed to another plate and the hemoglobin was monitored measuring the optical density at 550nm.

### β-Galactosidase assay

β-Galactosidase assays were performed as described [58]. Data are mean values of duplicate determinations in at least three independent experiments plotted with standard deviations.

### Expression analysis by qPCR

RNA was purified from three independent cultures grown in LB medium at 37°C up to an OD_600nm_ of 2.0 by using Total RNA Isolation kit (Promega) according to the manufacturer’s protocol. Samples were analyzed by Bioanalyzer 2100 from Agilent in order to verify RNA quality. After determination of the RNA concentrations using the NanoDropND-1000 V3.3.0 Spectrophotometer, 1 μg RNA was reverse transcribed in a 20 μL reaction using High-Capacity cDNA Reverse Transcription kit (Life Technologies). Primers used for quantification of the *hilA*, *hilD*, *hilC* and *rtsA* transcripts are listed in S2 Table. cDNA was diluted 1:100 and used as template in the real-time PCR reaction using SYBR Green PCR Master Mix X2 kit (Life Technologies). The cycling reaction was performed with a Step One Real-Time PCR system (Life Technologies). Individual gene expression profiles were normalized against the *gapA* gene (GAPDH) as endogenous control. In all experiments, the change in expression was measured relative to a WT strain, which was set to 1.0. The data values presented in all figures represent the mean values calculated from at least three independent experiments performed with three technical replicates. The error bars represent the standard deviations.

### Expression analysis by RT-PCR

The mRNA levels of *sipA* were monitored by RT-PCR using the Transcriptor One-Step RT-PCR Kit (Roche) and the primer pairs SipAFor/SipARev (S2 Table). The RT-PCR was carried out in a Bio-Rad T100 thermal cycler. First, the RNA was reversely transcribed for 15 min at 50°C, following by reverse transcriptase inactivation by incubation for 7 min at 94°C. The cDNA was amplified by 35 cycles of denaturation for 10 s at 94°C, annealing for 30 s at 54°C, and extension for 30 s at 68°C, with a final extension step of 7 min at 68°C. 16S rRNA was used as the internal control, using primers SalI16S/SalII16S (S2 Table). In all cases, the amount of total RNA used was defined by performing saturation curves with increasing amounts of total RNA to determine the interval of lineal increase in the relative amount of RT-PCR product and total RNA.

## Supporting information

S1 Fig Schematic representation of the regulatory pathway that control expression of the TTSS and effector proteins of the SPI-1 of *S*. Typhimurium. (PDF)

S2 Fig Invasion of epithelial cells by *S*. Typhimurium ATCC14028 is impaired in strains deficient for the Gre factors. Invasion assays used HT-29 epithelial cells. Cultures of the WT (UMR1) and the Δ*greA*, Δ*greB* and Δ*greA*Δ*greB* derivatives were assessed. As a control, cultures of the invasion impaired mutant Δ*motA* were used. A bar shows the arithmetic mean of experimental results and the error bar indicates the standard deviation. Significance was tested by an unpaired two–sided Student’s t-test. Statistical significance is indicated by **p<0.01, ns: non-significant.
(PDF)

S3 Fig Profile of secreted proteins in a Δ*hilA* derivative strain. Protein extracts from cell-free supernatants of two independent LB cultures of WT (SV5015) and its Δ*hilA* derivative. Extracts were analyzed by Coomassie blue stained 12.5% SDS-PAGE. Lane M: molecular mass markers (size in kDa indicated). The bands labelled were identified as SipA (1), FliD (2) and SipC (3) by LC-MS/MS.
(PDF)

S4 Fig The Δ*hilD*-cmR mutant strain is trans-complemented by a pBR322-based plasmid carrying the *hilD* gene. Cultures of the strains SV5015UB2 and TGC-10 were grown in LB at 37°C up to an OD_600nm_ of 2.0. Data are the average and error bars represent standard deviations from three biological replicates.
(PDF)

S5 Fig Ectopic induction of *hilD* expression elicited SPI-1 effector proteins even in the absence of the Gre factors. Cell-free supernatants of LB cultures of WT and Δ*greA*Δ*greB* strains carrying either pBAD18 or pBADHilD grown in LB at 37°C up to an OD_600nm_ of 2.0, arabinose (0.02%) was added in all cultures. Extracts were analyzed by Coomassie blue stained 12.5% SDS-PAGE.
(PDF)

S6 Fig Effect of the Gre factors on *Salmonella* swimming motility. Single colonies of the indicated strains were inoculated on either 0.3% LB agar plates (A) or 0.3% LB agar plates supplemented with 0.2% L-arabinose and 50 μg/ml of ampicillin (B). Plates were incubated at 37°C for 5 hours and swimming motility diameter was measured. A bar shows the arithmetic mean of experimental results and the error bar indicates the standard deviation from 5 replicates.
(PDF)

S7 Fig The absence of 3’-UTR of *hilD* causes a severe upregulation of the secreted SPI-1 effector protein levels even in the absence of Gre factors. Cell-free supernatants of LB cultures of WT and Δ*greA*Δ*greB* strains in both *hilD* 3’UTR+ and *hilD* 3’UTR- genetic backgrounds. Cultures were grown at 37°C up to an OD_600nm_ of 2.0. Extracts were analyzed by Coomassie blue stained 12.5% SDS-PAGE.
(PDF)

S8 Fig Representative control experiment for loading normalization of secreted extracts. A. Coomassie stained SDS-PAGE of either cell extracts (upper panel) or secreted protein extracts (lower panel) from two cultures of the strains SV5015 (WT) and TGC3 (Δ*greA*Δ*greB*) grown in LB at 37°C up to an OD_600nm_ of 2.0. Lane M: molecular mass markers (size in kDa indicated). B. Immunodetection of CRP, a cytoplasmic protein, in the indicated extracts from the same cultures as in A.
(PDF)

S1 Table Strains and plasmids used in this study. (PDF)

S2 Table Primers used in this study. (PDF)
