# Supplementary material for: Gre factors-mediated control of hilD transcription is essential for the invasion of epithelial cells by Salmonella enterica serovar Typhimurium
Source: PLoS Pathog. 2017 Apr 20;13(4):e1006312. doi: 10.1371/journal.ppat.1006312 (PMC5398713; doi:10.1371/journal.ppat.1006312)
Supplement: S1 Fig — (PDF) [file ppat.1006312.s001.pdf]

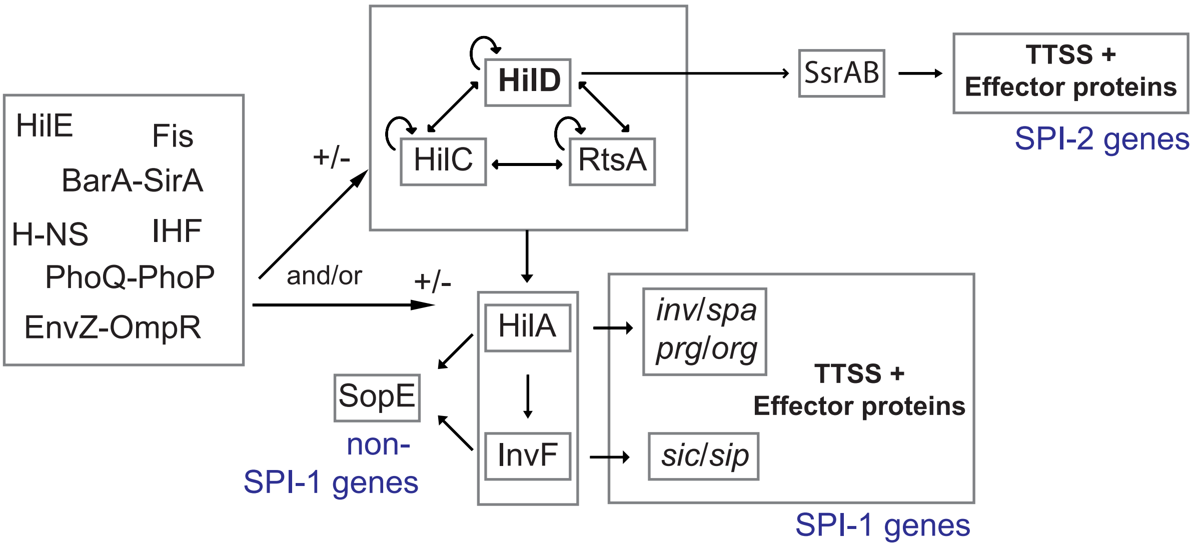

S1 Figure. **Schematic representation of the regulatory pathway that control expression of the TTSS and effector proteins of the SPI-1 of *S.* Typhimurium.**
