# Supplementary material for: Gre factors-mediated control of hilD transcription is essential for the invasion of epithelial cells by Salmonella enterica serovar Typhimurium
Source: PLoS Pathog. 2017 Apr 20;13(4):e1006312. doi: 10.1371/journal.ppat.1006312 (PMC5398713; doi:10.1371/journal.ppat.1006312)
Supplement: S2 Fig — Invasion assays used HT-29 epithelial cells. Cultures of the WT (UMR1) and the ΔgreA, ΔgreB and ΔgreAΔgreB derivatives were assessed. As a control, cultures of the invasion impaired mutant ΔmotA were used. A bar shows the arithmetic mean of experimental results and the error bar indicates the standard deviation. Significance was tested by an unpaired two–sided Student’s t-test. Statistical significance is indicated by **p<0.01, ns: non-significant. (PDF) [file ppat.1006312.s002.pdf]

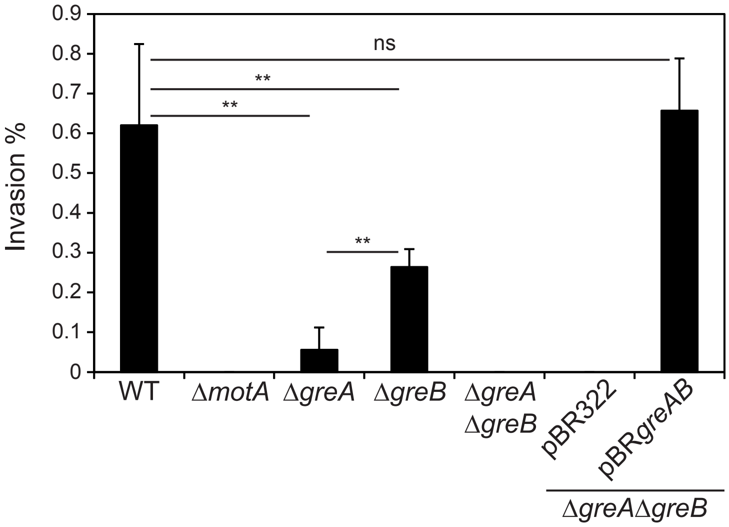

S2 Figure. Invasion of epithelial cells by *S*. Typhimurium ATCC14028 is impaired in strains deficient for the Gre factors. Invasion assays used HT-29 epithelial cells. Cultures of the WT (UMR1) and the Δ*greA*, Δ*greB* and Δ*greA*Δ*greB* derivatives were assessed. As a control, cultures of the invasion impaired mutant Δ*motA* were used. A bar shows the arithmetic mean of experimental results and the error bar indicates the standard deviation. Significance was tested by an unpaired two–sided Student’s t-test. Statistical significance is indicated by **$p<0.01$, ns: non-significant.
