# Supplementary material for: Gre factors-mediated control of hilD transcription is essential for the invasion of epithelial cells by Salmonella enterica serovar Typhimurium
Source: PLoS Pathog. 2017 Apr 20;13(4):e1006312. doi: 10.1371/journal.ppat.1006312 (PMC5398713; doi:10.1371/journal.ppat.1006312)
Supplement: S3 Fig — Protein extracts from cell-free supernatants of two independent LB cultures of WT (SV5015) and its ΔhilA derivative. Extracts were analyzed by Coomassie blue stained 12.5% SDS-PAGE. Lane M: molecular mass markers (size in kDa indicated). The bands labelled were identified as SipA (1), FliD (2) and SipC (3) by LC-MS/MS. (PDF) [file ppat.1006312.s003.pdf]

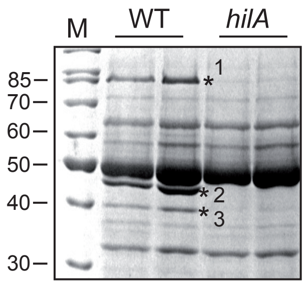

S3 Figure. **Profile of secreted proteins in a Δ*hilA* derivative strain.**
Protein extracts from cell-free supernatants of two independent LB cultures of WT (SV5015) and its Δ*hilA* derivative. Extracts were analyzed by Coomassie blue stained 12.5 % SDS-PAGE. Lane M: molecular mass markers (size in kDa indicated). The bands labelled were identified as SipA (1), FliD (2) and SipC (3) by LC-MS/MS.
