# Supplementary material for: Gre factors-mediated control of hilD transcription is essential for the invasion of epithelial cells by Salmonella enterica serovar Typhimurium
Source: PLoS Pathog. 2017 Apr 20;13(4):e1006312. doi: 10.1371/journal.ppat.1006312 (PMC5398713; doi:10.1371/journal.ppat.1006312)
Supplement: S4 Fig — Cultures of the strains SV5015UB2 and TGC-10 were grown in LB at 37°C up to an OD600nm of 2.0. Data are the average and error bars represent standard deviations from three biological replicates. (PDF) [file ppat.1006312.s004.pdf]

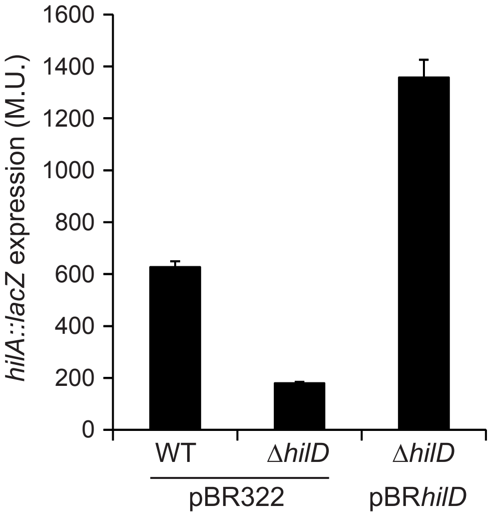

S4 Figure. The Δ*hilD*-cm^R mutant strain is trans-complemented by a pBR322-based plasmid carrying the *hilD* gene. Cultures of the strains SV5015UB2 and TGC-10 were grown in LB at 37ºC up to an OD_{600nm} of 2.0. Data are the average and error bars represent standard deviations from three biological replicates.
