# Supplementary material for: Gre factors-mediated control of hilD transcription is essential for the invasion of epithelial cells by Salmonella enterica serovar Typhimurium
Source: PLoS Pathog. 2017 Apr 20;13(4):e1006312. doi: 10.1371/journal.ppat.1006312 (PMC5398713; doi:10.1371/journal.ppat.1006312)
Supplement: S5 Fig — Cell-free supernatants of LB cultures of WT and ΔgreAΔgreB strains carrying either pBAD18 or pBADHilD grown in LB at 37°C up to an OD600nm of 2.0, arabinose (0.02%) was added in all cultures. Extracts were analyzed by Coomassie blue stained 12.5% SDS-PAGE. (PDF) [file ppat.1006312.s005.pdf]

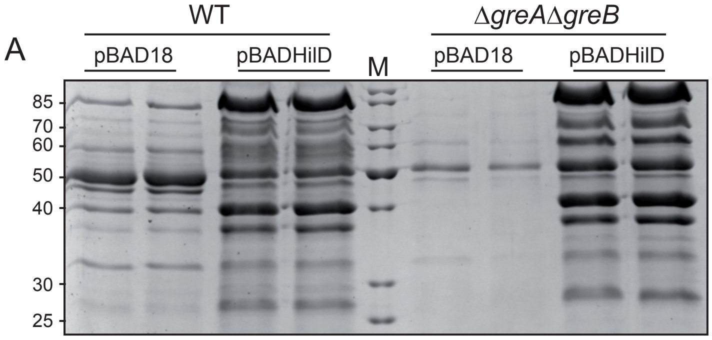

S5 Figure. Ectopic induction of *hilD* expression elicited SPI-1 effector proteins even in the absence of the Gre factors. Cell-free supernatants of LB cultures of WT and Δ*greA*Δ*greB* strains carrying either pBAD18 or pBADHilD grown in LB at 37ºC up to an OD_{600nm} of 2.0, arabinose (0.02 %) was added in all cultures. Extracts were analyzed by Coomassie blue stained 12.5 % SDS-PAGE.
