# Supplementary material for: Gre factors-mediated control of hilD transcription is essential for the invasion of epithelial cells by Salmonella enterica serovar Typhimurium
Source: PLoS Pathog. 2017 Apr 20;13(4):e1006312. doi: 10.1371/journal.ppat.1006312 (PMC5398713; doi:10.1371/journal.ppat.1006312)
Supplement: S6 Fig — Single colonies of the indicated strains were inoculated on either 0.3% LB agar plates (A) or 0.3% LB agar plates supplemented with 0.2% L-arabinose and 50 μg/ml of ampicillin (B). Plates were incubated at 37°C for 5 hours and swimming motility diameter was measured. A bar shows the arithmetic mean of experimental results and the error bar indicates the standard deviation from 5 replicates. (PDF) [file ppat.1006312.s006.pdf]

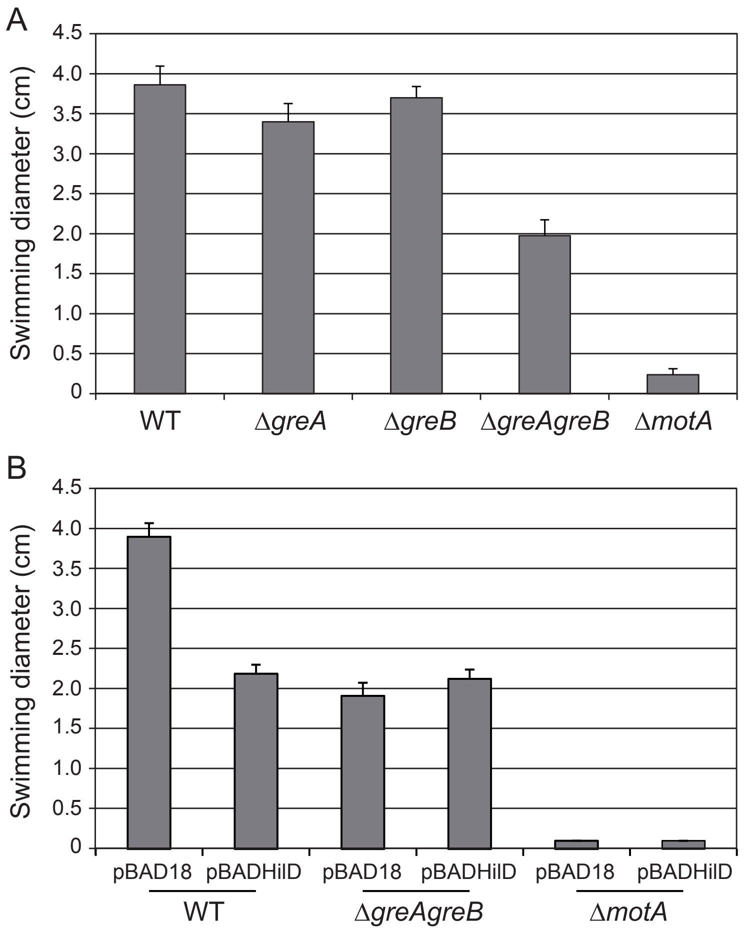

S6 Figure. Effect of the Gre factors on *Salmonella* swimming motility. Single colonies of the indicated strains were inoculated on either 0.3% LB agar plates (A) or 0.3% LB agar plates supplemented with 0.2% L-arabinose and 50 µg/ml of ampicillin (B). Plates were incubated at 37ºC for 5 hours and swimming motility diameter was measured.
A bar shows the arithmetic mean of experimental results and the error bar indicates the standard deviation from 5 replicates.
