# Supplementary material for: Gre factors-mediated control of hilD transcription is essential for the invasion of epithelial cells by Salmonella enterica serovar Typhimurium
Source: PLoS Pathog. 2017 Apr 20;13(4):e1006312. doi: 10.1371/journal.ppat.1006312 (PMC5398713; doi:10.1371/journal.ppat.1006312)
Supplement: S7 Fig — Cell-free supernatants of LB cultures of WT and ΔgreAΔgreB strains in both hilD 3’UTR+ and hilD 3’UTR- genetic backgrounds. Cultures were grown at 37°C up to an OD600nm of 2.0. Extracts were analyzed by Coomassie blue stained 12.5% SDS-PAGE. (PDF) [file ppat.1006312.s007.pdf]

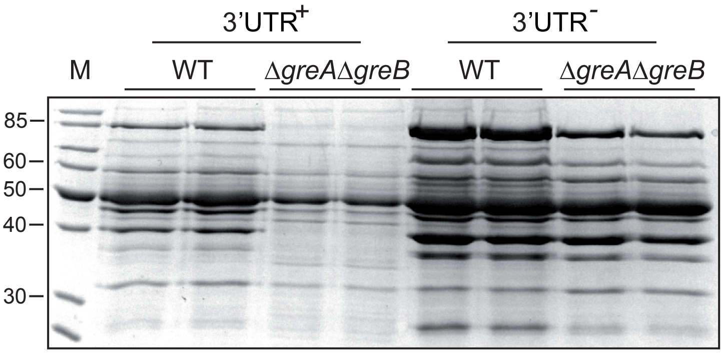

S7 Figure. The absence of 3'-UTR of *hilD* causes a severe upregulation of the secreted SPI-1 effector protein levels even in the absence of Gre factors. Cell-free supernatants of LB cultures of WT and Δ*greA*Δ*greB* strains in both *hilD*3'UTR+ and *hilD*3'UTR- genetic backgrounds. Cultures were grown at 37ºC up to an OD_{600nm} of 2.0. Extracts were analyzed by Coomassie blue stained 12.5 % SDS-PAGE.
