# Supplementary material for: Gre factors-mediated control of hilD transcription is essential for the invasion of epithelial cells by Salmonella enterica serovar Typhimurium
Source: PLoS Pathog. 2017 Apr 20;13(4):e1006312. doi: 10.1371/journal.ppat.1006312 (PMC5398713; doi:10.1371/journal.ppat.1006312)
Supplement: S8 Fig — A. Coomassie stained SDS-PAGE of either cell extracts (upper panel) or secreted protein extracts (lower panel) from two cultures of the strains SV5015 (WT) and TGC3 (ΔgreAΔgreB) grown in LB at 37°C up to an OD600nm of 2.0. Lane M: molecular mass markers (size in kDa indicated). B. Immunodetection of CRP, a cytoplasmic protein, in the indicated extracts from the same cultures as in A. (PDF) [file ppat.1006312.s008.pdf]

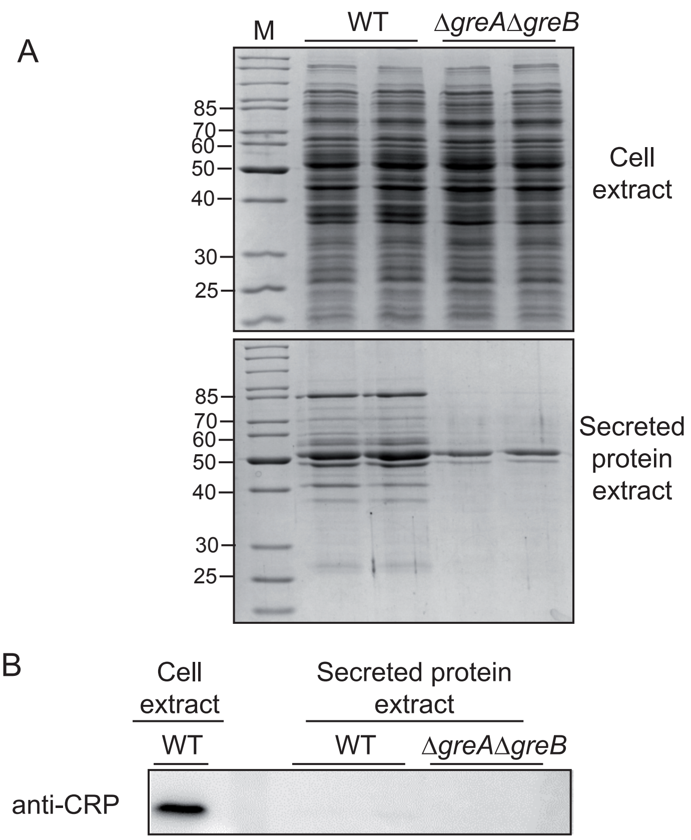

S8 Figure. Representative control experiment for loading normalization of secreted extracts.
A. Coomassie stained SDS-PAGE of either cell extracts (upper panel) or secreted protein extracts (lower panel) from two cultures of the strains SV5015 (WT) and TGC3 (Δ*greA*Δ*greB*) grown in LB at 37ºC up to an OD_{600nm} of 2.0. Lane M:molecular mass markers (size in kDa indicated).
B. Immunodetection of CRP, a cytoplasmic protein, in the indiacted extracts from the same cultures as in A.
