# Supplementary material for: Gre factors-mediated control of hilD transcription is essential for the invasion of epithelial cells by Salmonella enterica serovar Typhimurium
Source: PLoS Pathog. 2017 Apr 20;13(4):e1006312. doi: 10.1371/journal.ppat.1006312 (PMC5398713; doi:10.1371/journal.ppat.1006312)
Supplement: S1 Table — (PDF) [file ppat.1006312.s009.pdf]

S1 Table. Strains and plasmids used in this study

Strain	Relevant characteristics or description	Reference
SV5015	*Salmonella enterica* serovar Typhimurium SL1344 *his*$^{+}$	[1]
TGC-1	SV5015 *greA*	This study
TGC-2	SV5015 *greB*	This study
TGC-3	SV5015 *greAgreB*	This study
TGC-65	SV5015 *greA*-km^{R} *greB*	This study
SV5293	14028 *sipC::lacZ*-km^{R} (Δ 76 to 1112)	[2]
TGC-40	SV5015 *sipC::lacZ*-km^{R}	This study.
TGC-41	SV5015 *greAgreB sipC::lacZ*-km^{R}	This study
MHS-1	SV5015 *hilA*	[3]
MHQ-2	SV5015 *hilC*	[3]
TGC-4	SV5015 *hilD*-cm^{R}	This study
TGC-6	SV5015 *rtsA*-cm^{R}	This study
SV5015UB2	SV5015 *hilA::lacZ*-km^{R} (Δ 44 to 1621)	[4]
TGC-7	SV5015 *greAgreB hilA::lacZ*-km^{R}	This study
TGC-8	SV5015 *hilC hilA::lacZ*-km^{R}	This study
TGC-9	SV5015 *hilC greAgreB hilA::lacZ*-km^{R}	This study
TGC-10	SV5015 *hilD*-cm^{R} *hilA::lacZ*-km^{R}	This study
TGC-11	SV5015 *greAgreB hilD*-cm^{R} *hilA::lacZ*-km^{R}	This study
TGC-14	SV5015 *rtsA*-cm^{R} *hilA::lacZ*-km^{R}	This study
TGC-15	SV5015 *greAgreB rtsA*-cm^{R} *hilA::lacZ*-km^{R}	This study
MHS-2	SV5015 *sipA::*3Flag-km^{R}	[5]
TGC-16	SV5015 *greAgreB sipA::*3Flag-km^{R}	This study
JPTM7	SV5015 *hilA::*3Flag-km^{R}	[6]
TGC-17	SV5015 *greAgreB hilA::*3Flag-km^{R}	This study
TGC-18	SV5015 *hilD*-cm^{R} *hilA::*3Flag-km^{R}	This study
TGC-19	SV5015 *greAgreB hilD*-cm^{R} *hilA::*3Flag-km^{R}	This study

TGC-20	SV5015 *invF::*3Flag-km^R	This study
TGC-21	SV5015 *greAgreB invF::*3Flag-km^R	This study
JPTM8	SV5015 *ssrA::*3Flag-km^R	[6]
TGC-22	SV5015 *hilD*-cm^R *ssrA::*3Flag-km^R	This study
TGC-23	SV5015 *greAgreB ssrA::*3Flag-km^R	This study
TGC-24	SV5015 *greAgreB hilD*-cm^R *ssrA::*3Flag-km^R	This study
TGC-25	SV5015 $hilD_{+76}$*::lacZ*-km^R	This study
TGC-26	SV5015 *greAgreB* $hilD_{+76}$*::lacZ*-km^R	This study
TGC-35	SV5015 $hilD_{+1235}$*::lacZ*-km^R	This study
TGC-36	SV5015 *greAgreB* $hilD_{+1235}$*::lacZ*-km^R	This study
TGC-38	SV5015 $hilD_{+965}$*::lacZ*-km^R	This study
TGC-39	SV5015 *greAgreB* $hilD_{+965}$*::lacZ*-km^R	This study
SV6190	14028 *hilD*Δ3'UTR-km^R	[7]
TGC-53	SV5015 *hilD*Δ3'UTR-km^R	This study
TGC-37	SV5015 *greAgreB hilD*Δ3'UTR	This study
TGC-54	SV5015 *hilD*Δ3'UTR *sipC::lacZ*-km^R	This study
TGC-55	SV5015 *greAgreB hilD*Δ3'UTR *sipC::lacZ*-km^R	This study
SV6212	14028 *araBAD*-cm^R *hilA::lacZ*-km^R	[8]
SV6197	14028 *araC*-km^R	[8]
TGC-61	SV5015 *araBAD*-cm^R *araC*-km^R	This study
TGC-62	SV5015 *greAgreB araBAD*-cm^R *araC*-km^R	This study
TGC-66	SV5015 *hfq*-cm^R	This study
TGC-67	SV5015 *greAgreB hfq*-cm^R	This study
SV5961	14028 *rne*-cm^R (*rne537*)	[2]
TGC-68	SV5015 *rne*-cm^R	This study
TGC-69	SV5015 *greAgreB rne*-cm^R	This study
TGC-42	SV5015 *motA*	This study
TT1704	Δ*his*-9533	[9]

UMR1	ATCC 14028 nal^{R}	[10]
LB5000	*rLT*$^{-}$ *rSA*$^{-}$ *rSB*$^{-}$	[11]

**Plasmids**	**Relevant characteristics or description**	**Reference**
pKD3	*bla* FRT cm^{R} PS1 PS2 oriR6K	[12]
pKD4	*bla* FRT km^{R} PS1 PS2 oriR6K	[12]
pKD46	*bla* P$_{BAD}$ *gam bet exo* pSC101 oriTS	[12]
pCP20	*bla* cm^{R} *cI*857 λP$_{R}$ *flp* pSC101 oriTS	[12]
pKG137	*ahp* FRT *lacZY*+ t$_{his}$, oriR6K	[13]
pKG136	*ahp* FRT *lacZY*+ t$_{his}$, oriR6K	[13]
pSUB11	3xFLAG- and Km^{R}-coding template vector	[14]
pGEM-T easy	oripMB1 Cb^{R}	[15]
pBAD18	p*ara*BAD oripMB1Amp^{R}	[16]
pBADHilA	pBAD18 + *hilA* ORF $_{SV5015}$	[5]
pBADHilD	pBAD18 + *hilD* ORF $_{SV5015}$	This study
pBR322	oripMB1 Tc^{R}, Amp^{R}	[17]
pBR*greA*	pBR322+*greA*$_{SV5015}$	This study
pBR*greB*	pBR322+*greB*$_{SV5015}$	This study
pBR*greAB*	pBR322+*greAgreB*$_{SV5015}$	This study
pBR*hilD*	pBR322+*hilD*$_{SV5015}$	This study
pHM1883	Ptrc expression vector, oripGB2 Spec^{R}	[18]
pHM1873	pHM1883+*greA*$_{MG1655}$	[18]
pHM1854	pHM1883+*greA* $_{MG1655}$ (D41A E44Y)	[18]
pTT68	P$_{BAD}$-MCS-*lacZ*, oriRO1600/MB1 Amp^{R}	This study
pTTORF	pTT+ *hilD* ORF	This study
pTT3'UTR	pTT+ *hilD* 3'UTR	This study
pTT3'UTR+T	pTT+ *hilD* 3'UTR+Terminator	This study

References

1. Vivero A, Baños RC, Mariscotti JF, Oliveros JC, García-del Portillo F, Juárez A, et al. Modulation of horizontally acquired genes by the Hha-YdgT proteins in *Salmonella enterica* serovar Typhimurium. J Bacteriol. 2008;190: 1152–1156.
2. López-Garrido J, Casadesús J. Regulation of *Salmonella enterica* pathogenicity island 1 by DNA adenine methylation. Genetics. 2010;184: 637–649.
3. Queiroz MH, Madrid C, Paytubi S, Balsalobre C, Juarez A. Integration host factor alleviates H-NS silencing of the *Salmonella enterica* serovar Typhimurium master regulator of SPI1, *hilA*. Microbiology. 2011;157: 2504–2514.
4. Baños RC, Vivero A, Aznar S, García J, Pons M, Madrid C, et al. Differential regulation of horizontally acquired and core genome genes by the bacterial modulator H-NS. PLoS Genet. 2009;5: e1000513.
5. Hüttener M, Dietrich M, Paytubi S, Juárez A. HilA-like regulators in *Escherichia coli* pathotypes: the YgeH protein from the enteroaggregative strain 042. BMC Microbiol. 2014;14: 268.
6. Bustamante VH, Martínez LC, Santana FJ, Knodler LA, Steele-Mortimer O, Puente JL. HilD-mediated transcriptional cross-talk between SPI-1 and SPI-2. Proc Natl Acad Sci U S A. 2008;105: 14591–14596.
7. López-Garrido J, Puerta-Fernández E, Casadesús J. A eukaryotic-like 3’ untranslated region in *Salmonella enterica hilD* mRNA. Nucleic Acids Res. 2014;42: 5894–5906.
8. López-Garrido J, Puerta-Fernández E, Cota I, Casadesús J. Virulence Gene Regulation by L-Arabinose in *Salmonella enterica*. Genetics. 2015;200: 807–819.
9. Torreblanca J, Casadesús J. DNA adenine methylase mutants of *Salmonella typhimurium* and a novel dam-regulated locus. Genetics. 1996;144: 15–26.
10. Römling U, Bian Z, Hammar M, Sierralta WD, Normark S. Curli fibers are highly conserved between *Salmonella typhimurium* and *Escherichia coli* with respect to operon structure and regulation. J Bacteriol. 1998;180: 722–731.
11. Bullas LR, Ryu JI. *Salmonella typhimurium* LT2 strains which are r- m+ for all three chromosomally located systems of DNA restriction and modification. J Bacteriol. 1983;156: 471–474.
12. Datsenko KA, Wanner BL. One-step inactivation of chromosomal genes in *Escherichia coli* K-12 using PCR products. Proc Natl Acad Sci U S A. 2000;97: 6640–6645.
13. Ellermeier CD, Janakiraman A, Slauch JM. Construction of targeted single copy *lac* fusions using lambda Red and FLP-mediated site-specific recombination in bacteria. Gene. 2002;290: 153–161.
14. Uzzau S, Figueroa-Bossi N, Rubino S, Bossi L. Epitope tagging of chromosomal genes in *Salmonella*. Proc Natl Acad Sci U S A. 2001;98: 15264–15269.
15. Yanisch-Perron C, Vieira J, Messing J. Improved M13 phage cloning vectors and host strains: nucleotide sequences of the M13mp18 and pUC19 vectors. Gene. 1985;33: 103–119.

16. Guzman LM, Belin D, Carson MJ, Beckwith J. Tight regulation, modulation, and high-level expression by vectors containing the arabinose PBAD promoter. J Bacteriol. 1995;177: 4121–4130.

17. Bolivar F, Rodriguez RL, Greene PJ, Betlach MC, Heyneker HL, Boyer HW, et al. Construction and characterization of new cloning vehicles. II. A multipurpose cloning system. Gene. 1977;2: 95–113.

18. Vinella D, Potrykus K, Murphy H, Cashel M. Effects on growth by changes of the balance between GreA, GreB, and DksA suggest mutual competition and functional redundancy in *Escherichia coli*. J Bacteriol. 2012;194: 261–273.
