# Supplementary material for: Gre factors-mediated control of hilD transcription is essential for the invasion of epithelial cells by Salmonella enterica serovar Typhimurium
Source: PLoS Pathog. 2017 Apr 20;13(4):e1006312. doi: 10.1371/journal.ppat.1006312 (PMC5398713; doi:10.1371/journal.ppat.1006312)
Supplement: S2 Table — (PDF) [file ppat.1006312.s010.pdf]

S2 Table. Primers used in this study

Name	Sequence (5' to 3')
	**One-step inactivation of chromosomal genes**
**greAP1**	CCCTACAGGAATGTTCAAGAGGTATAACAAATGCAAGCTGTGTAGGCTGG AGCTGCTTC
**greAP2**	TACACCAACAATTTGCGTATTGAGTACTGCTTAAAGGTACATATGAATATC CTCCTTAGT
**greAP1UP**	TGACCCTGGTATGGCATAT
**greAP2DOWN**	TCTGTGTAAAACGAGGGGTT
**greBP1**	CAATATCGACAGCAAAGGTAAATCAACGAGATGAAAACGGTGTAGGCTGG AGCTGCTTC
**greBP2**	CATCAGCGGGGGCTTAGGATTCTTCTTGTCTTATTTGACCATATGAATATC CTCCTTAGT
**greBP1UP**	CTTAAATATACAATTAATCGGC
**greBP2DOWN**	AAAATCAGGGGATAGTTATAC
**hilDP1+76**	ATGGAAAATGTAACCTTTGTAAGTAATAGTCATCAGCGTCCTGTGTAGGCT GGAGCTGCTTC
**hilDP2.1**	TTAATGGTTCGCCATTTTTATGAATGTCGATGGCGTAGTTTTCATATGAATA TCCTCCTTAGT
**hilDP1UP**	GTAGGATACCAGTAAGGAAC
**hilDP2DOWN**	GCGTGTTAATGCGCAGTCTG
**hilDP1+1235**	GATATTGCCTTATTCACATCGTAAGAATTCGTCCAGATGACACTATCTCCG TGTAGGCTGGAGCTGCTTC
**hilDP2.2**	TATAAATATGAATAAAATGCCGGCCTTAATCCACAGGGTTAAAGCCGGAA CATATGAATATCCTCCTTAGT
**rtsAP1**	GCACATTTAATAAAAGGAAATTATCATGCTAAAAGTATTTAATCCCTCACC GTGTAGGCTGGAGCTGCTTC
**rtsAP2**	TCTTATACTGCATTGTCAGATATCTCAATTAACATATTGATGACGAGAGGC ATATGAATATCCTCCTTAGT
**Hfq-P1**	GTACAATTGAGACGTATCGTGCGCAATTTTTCAGAATCGAGTGTAGGCTG GAGCTGCTTC
**Hfq-P2**	CCCGACATGGATAAACAGCGCGTGAACTTATTCAGTCTCTTGATATGAATA TCCTCCTTA
**rtsAP1UP**	GATCGCCACCTGATACCTTA
**rtsAP2DOWN**	GCGAGCAACAGAATCCCATC
**KT**	CGGCCACAGTCGATGAATCC
**K2**	CGGTGCCCTGAATGAACTGC
**C1**	CCTTGTCGCCTTGCGTATAA
**C2**	CCTACCTGTGACGGAAGATC
**LACZR**	GATGACCTGCAAGGCGATTA

	**Epitope tagging of chromosomal genes in *Salmonella***
**InvFP13Flag**	GCCGCGGAAATTATCAAATATTATTCAATTGGCAGACAAAGACTACAAAGACCATGACGG
**InvFP23Flag**	GCGGCACATGCCAGCACTCTGGCCAAAAGAATATGTGTCTCATATGAATATCCTCCTTAG
**InvFP13FlagUP**	GCGTATGGCGCAATCGCTGC
**InvFP23FlagDOWN**	CCCACTTCCCGTTACAGG
	**Cloning**
**greASalmUP**	GAATTCTCGCGCTAACACCCTGG
**greASalmDOWN**	GGATCCTCGCTGCCGCGTTAAGG
**greBSalmUP**	GAATTCGTACTCCCAAAGGTTCGC
**greBSalmDOWN**	GGATCCATGGGTTAGCTTCGTC
**greASalIUP**	GGGGTCGACGGAACTCCAGGGTAAAATGG
**greABamHIDOWN**	GGGGGATCCTCGCTGCCGCGTTAAGG
**pBR-FW**	CCATTATTATCATGAACATTAAC
**pBR-RV**	GATGCCGGCCACGATGCGTCC
**hilDNcoI1**	GGGCCATGGGGAGCGCGTTTACAACATTATA
**UTRSalI3**	GGGTCGACTTAAAATATTTTTTGAAACA
**UTRSalI8**	GGGTCGACGCAAATAGTTCTCAGAGGGAAC
**UTRSalI6**	GGGTCGACAAGGAGATAGTGTCATCTGGAC
**hilDFw322**	GGATCCTGTTAGCGATGTCTGTCG
**hilDRv322A**	GTCGACTGCCTGGCAGAAACTAAC
**hilDBADFw**	CGGAATTCTAACATCAACAAAGGGATAATATGGAA
**hilDBADRv**	GCTCTAGATTAATGGTTCGCCATTTTTATGAA
	* Restriction sites underlined
	**Quantitative PCR (qPCR)**
**hilDRTFW**	GCCAGAAGAG AGGTATTTG
**hilDqPCRRV**	CAGTAAGCAGGAACAGCAG
**hilAqPCR1**	GGATATTCTTGAGCTCATGG
**hilAqPCR2**	GAGAAGCGGGTTGGTGTTC
**hilCqPCR1**	CTCACCCGCAAATGGTCAC
**hilCqPCR2**	GCCTGATTCATACGAGCATC
**rtsART-FW**	GTATATTACGGCATCAGGGC
**rtsART-RV**	GCCTGTTTCTATTGGCGC
**GAPDHqPCR1**	GTCCGTCTAAAGACAACACC
**GAPDHqPCR2**	CATCAGACCTTCGATGATGC
	**RT-PCR**
**SipAFor**	GAACGGTGTGGAGGTATCTG
**SipARev**	GAGAATGTTAAAACCGATACC
**SalI16S**	CTACTGGAAACGGTGGCTAA
**SalII16S**	AAGCCTGCCAGTTTCGAATG
